# Structural and biophysical aspects of l-asparaginases: a growing family with amazing diversity

**DOI:** 10.1107/S2052252521006011

**Published:** 2021-06-30

**Authors:** Joanna I. Loch, Mariusz Jaskolski

**Affiliations:** a Department of Crystal Chemistry and Crystal Physics, Faculty of Chemistry, Jagiellonian University, Cracow, Poland; b Department of Crystallography, Faculty of Chemistry, A. Mickiewicz University, Poznan, Poland; c Center for Biocrystallographic Research, Institute of Bioorganic Chemistry, Polish Academy of Sciences, Poznan, Poland

**Keywords:** leukemia, l-asparaginases, amidohydrolases, catalytic mechanism, active site, nucleophiles

## Abstract

l-Asparaginases have remained an intriguing research topic since their discovery ∼120 years ago, especially after their introduction in the 1960s as very efficient antileukemic drugs. In addition to bacterial asparaginases, which are still used to treat childhood leukemia, enzymes of plant and mammalian origin are now also known. They have all been structurally characterized by crystallography, in some cases at outstanding resolution. The structural data have also shed light on the mechanistic details of these deceptively simple enzymes. Yet, despite all this progress, no better therapeutic agents have been found to beat bacterial asparaginases. However, a new option might arise with the discovery of yet another type of asparaginase, those from symbiotic nitrogen-fixing Rhizobia, and with progress in the protein engineering of enzymes with desired properties. This review surveys the field of structural biology of l-asparaginases, focusing on the mechanistic aspects of the well established types and speculating about the potential of the new members of this amazingly diversified family.

## Abstract 2

The structural and biophysical aspects of a diversified group of enzymes that hydrolyze l-asparagine are reviewed in the context of their mechanism and biological function, and as therapeutic agents for the treatment of leukemia.

## 1. Asparaginases: historical facts and structure-based classification   

**Figure 1:**
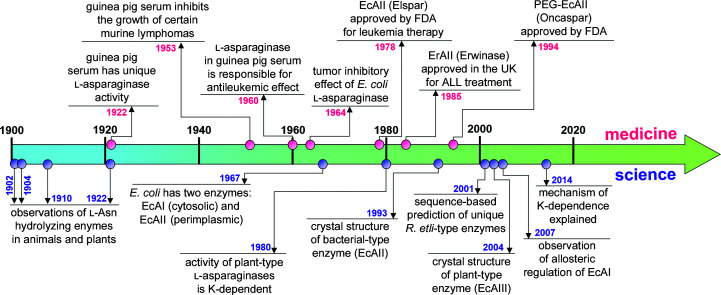
Overview of selected events in the history of l-asparaginases in science and medicine. Scientific discoveries intertwine with medical applications of l-­asparaginases. References to the historical facts presented are included in the text.

Asparaginases (EC 3.5.1.1) are amidohydrolases that catalyze hydrolysis at the side chain of l-asparagine to release l-aspartate and ammonia. This humble chemical reaction contrasts with the picturesque history of discovery of this amazingly diverse group of enzymes, which starts at the beginning of the 20th century with the observation of the existence of amide-splitting enzymes in suspensions of animal and plant tissues (Gonnermann, 1902 ▸). However, these early observations did not refer directly to the hydrolysis of l-asparagine (Geddes & Hunter, 1928 ▸; Fig. 1 ▸). The presence of enzymes hydrolyzing l-Asn (and l-Gln) was noticed in suspensions of beef liver (Lang, 1904 ▸), and horse and pig tissues (Furth & Friedmann, 1910 ▸). The serendipitous detection of the strong antileukemic properties of guinea pig serum (Kidd, 1953 ▸) was attributed by Broome to l-asparaginase activity (Broome, 1961 ▸), which had already been reported in guinea pig serum by Clementi (1922 ▸). The large-scale use of guinea pig serum in leukemia treatment was not possible, but in 1964 a similar tumor inhibitory effect was reported for *Escherichia coli* asparaginase (Mashburn & Wriston, 1964 ▸). These discoveries opened an era of intensive biochemical and structural studies of l-asparaginases, as l-Asn is vital for the survival of some cancer cells (Luo *et al.*, 2018 ▸). Most malignant lymphoblasts are unable to produce endogenous l-Asn, and in consequence these cancer cells are solely dependent on extracellular l-Asn for survival (Abaji & Krajinovic, 2019 ▸). Therapeutic l-asparaginases work by depleting the pool of circulating l-Asn, leading to cancer cell starvation and death (Boyse *et al.*, 1967 ▸; Prager & Bachynsky, 1968 ▸; Radadiya *et al.*, 2020 ▸). The requirement for a low *K*_m_ (at least micromolar) of this unusual enzymatic cancer drug stems from the fact that the intravenous l-asparaginase must be able to clear the circulating pools of l-Asn, which are not very high (about 50 µ*M*) (Ollenschläger *et al.*, 1988 ▸; Beckett & Gervais, 2019 ▸). The first therapeutic l-asparaginase (Elspar, EcAII) was approved by the US Food and Drug Administration in 1978. An improved, PEGylated version of EcAII (Oncaspar) with a prolonged circulation time and lower immunogenicity was approved in 1994 (Dinndorf *et al.*, 2007 ▸; Nunes *et al.*, 2020 ▸). For patients developing silent inactivation or strong allergic reactions to EcAII, an alternative bacterial enzyme derived from *Erwinia chrysanthemi* 1 (Erwinase, ErAII) was authorized in the UK in 1985 and in the USA in 2011 (Gervais *et al.*, 2013 ▸; Porton Biopharma, 1985 ▸).

**Figure 2:**
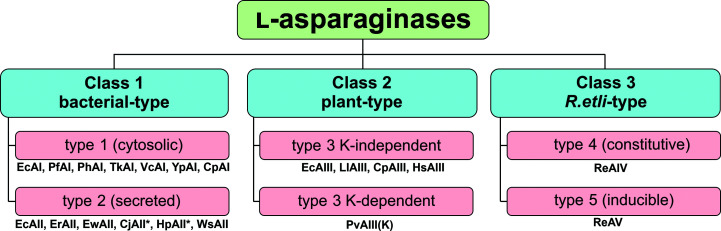
Structure-based classification of l-asparaginases. Historically, l-asparaginases were named according to the source organism of the first enzymes that were discovered. The new nomenclature divides l-asparaginases into three classes. Cytosolic type 1 enzymes are expressed constitutively, while the expression of type 2 enzymes, which are secreted to the periplasm, is induced under anaerobic conditions in *E. coli*. In the *R. etli*-type Class 3, constitutive type 4 enzymes are thermostable, while type 5 enzymes are considered thermolabile and their expression is induced by the presence of l-Asn. Examples of enzymes are listed below the boxes. The organism name abbreviations are as follows: Ec, *Esherichia coli*; Pf, *Pyrococcus furiosus*; Ph, *Pyrococcus horikoshii;* Tk, *Thermococcus kodakarensis*; Vc, *Vibrio cholerae*; Yp, *Yersinia pestis*; Cp, *Cavia porcellus*; Er, *Erwinia chrysanthemi*; Ew, *Erwinia carotovora*; Cj, *Campylobacter jejuni*; Hp, *Helicobacter pylori*; Ws, *Wolinella succinogenes*; Ll, *Lupinus luteus*; Pv, *Phaseolus vulgaris*; Hs, *Homo sapiens*; Re, *Rhizobium etli*. Examples of PDB codes are summarized in Supplementary Table S1. An asterisk denotes enzymes that are annotated as products of an *ansA* gene; however, the architecture of the active site and substrate affinity suggest classification as Class 1 type 2 enzymes.

In general, l-asparaginases are divided into three structural classes: bacterial-type, plant-type (Michalska *et al.*, 2006 ▸) and *Rhizobium etli*-type (Borek & Jaskólski, 2001 ▸) (Fig. 2 ▸). However, this classification, which is based on the sources of the first enzymes that were discovered, may be misleading (see Section 2). In fact, enzymes from all structural classes are distributed among different taxonomic groups, including microorganisms (for example bacteria and fungi), higher plants and animals (Borek *et al.*, 2004 ▸; Borek & Jaskólski, 2001 ▸). The bacterial-type enzymes can further be divided into two subclasses. This classification was initially introduced for the *E. coli* enzymes and was subsequently propagated to other proteins of similar architecture. *E. coli* possesses two enzymes in this class, encoded by the *ansA* and *ansB* genes. The protein products of these genes are termed EcAI (type 1, cytosolic, low-affinity enzyme) and EcAII (type 2, secreted, high-affinity enzyme), respectively. EcAI is expressed constitutively in the cytoplasm, while EcAII is expressed under anaerobic conditions in the periplasmic space (Campbell *et al.*, 1967 ▸). The *ansB* gene is not organized in any operon, while its expression is regulated by cyclic AMP receptor and fumarate and nitrate reductase regulation (FNR) proteins (Jennings & Beacham, 1990 ▸). Interestingly, the eukaryotic yeast *Saccharomyces cerevisiae* also has two asparaginases: a cytoplasmic aspara­ginase and a secreted asparaginase (Dunlop & Roon, 1975 ▸; Dunlop *et al.*, 1978 ▸). The first pieces of structural information, describing single crystals and the low-resolution structures of bacterial-type asparaginases (or their close glutaminase-asparaginase, G-A, homologs), were published between 1969 and 1988 (North *et al.*, 1969 ▸; Epp *et al.*, 1971 ▸; Lee & Yang, 1973 ▸; Wlodawer *et al.*, 1977 ▸; Yonei *et al.*, 1977 ▸; Ammon *et al.*, 1985 ▸, 1988 ▸; Tanaka *et al.*, 1988 ▸;). The first high-resolution crystal structure of EcAII (Elspar) with serendipitously bound l-aspartate was published in 1993^2^ (Swain *et al.*, 1993 ▸). It revealed the location of the active site and suggested a simple catalytic apparatus consisting of a Thr–Lys–Asp triad, reminiscent of the Ser–His–Asp triad of classic serine proteases (Seemüller *et al.*, 1996 ▸).

In the course of studies of l-asparagine (Atkins *et al.*, 1975 ▸) and nitrogen metabolism in symbiotic nitrogen-fixing legumes (Lees & Blakeney, 1970 ▸) such as lupins (Lea *et al.*, 1978 ▸), it was discovered that plants possess potassium-dependent and potassium-independent asparaginases (Sodek *et al.*, 1980 ▸). However, the molecular mechanism of their potassium dependence was only explained in 2014 on the basis of crystallographic studies (Bejger *et al.*, 2014 ▸). The cDNA encoding lupin l-asparaginase revealed that this enzyme differs from bacterial-type l-asparaginases but is similar to human aspartyl­glucosaminidase (Lough *et al.*, 1992 ▸). A few years later, human aspartylglucosaminidase (also termed, incorrectly, glycosylasparaginase), together with other amido­hydrolases, was classified as an N-terminal nucleophile hydrolase (Ntn-hydrolase; Brannigan *et al.*, 1995 ▸). Biochemical studies suggested that asparaginases from higher plants also belong to the Ntn-hydrolases (Oinonen & Rouvinen, 2000 ▸; Hejazi *et al.*, 2002 ▸). This hypothesis was confirmed by the first crystal structure to be determined of a plant-type l-asparaginase, that of LlA from yellow lupin (*Lupinus luteus*; Michalska *et al.*, 2006 ▸). Surprisingly, a gene (*yibK*) encoding a plant-type asparaginase was also found in the *E. coli* genome, and the protein product of this gene, with 70% sequence similarity to LlA, was termed EcAIII (Borek & Jaskólski, 2000 ▸). The crystal structure of EcAIII confirmed its classification as an Ntn-hydrolase (Borek & Jaskólski, 2000 ▸; Prahl *et al.*, 2004 ▸; Michalska *et al.*, 2005 ▸). Plant-type (or type 3) l-asparaginases are also capable of hydrolyzing β-aspartyl peptides and this isoaspartyl aminopeptidase activity can even dominate (Borek *et al.*, 2004 ▸).

Studies of *R. etli*, a nitrogen-fixing bacterium that associates symbiotically with the legume *Phaseolus vulgaris*, revealed that it possesses two types of asparaginase: a thermostable asparaginase and a thermolabile asparaginase (Huerta-Zepeda *et al.*, 1997 ▸). Most intriguingly, a comprehensive phylogenetic analysis of l-asparaginase sequences showed that the *R. etli* enzymes belong to a completely different class of proteins, significantly distant from the bacterial-type and plant-type enzymes (Borek & Jaskólski, 2001 ▸). For this reason, a new class of asparaginases was established early on, but its structural confirmation has only just begun to emerge (Loch *et al.*, unpublished work).

## 2. l-Asparaginase nomenclature issues   

Historically, enzymes with asparaginase activity were named according to the source organism, with the possible addition of a Roman numeral (I, II *etc.*) when more than one enzyme had a similar origin. Thus, we have the *E. coli* cytoplasmic EcAI and periplasmic EcAII asparaginases. The first organism in which a representative of a new structural class was discovered was then used to distinguish a family or type of asparaginases. Thus, the above two *E. coli* enzymes are bacterial-type. A terminological complication arose when it was realized that enzymes typical of one kingdom (for example plant-type) are also found in other kingdoms, as illustrated by EcAIII. In want of a better system, such proteins were still classified with the original type; this is why EcAIII is called plant-type. This has led to a Babel of nomenclature because enzymes of the three canonical types, *i.e.* bacterial-type, plant-type and *R. etli*-type, are widely found throughout several kingdoms of life. To rectify the situation, Schultz da Silva *et al.* (2021 ▸) have recently proposed that the class name should be detached from the biological taxonomy, and to use Class 1, Class 2 and Class 3 with reference to the three canonical classes above. We will use this reasonable proposal in our review, hoping that it will be universally adopted in the field. However, we do not feel that this change of global terminology should be transmitted down to concrete enzymes, which have historically sanctioned names that are firmly established in the literature. Thus, we will say that EcAI belongs to type 1 of the Class 1 aspara­ginases. Analogously, EcAII is type 2 in the same Class 1, while EcAIII is a type 3 asparaginase in structural Class 2. The *R. etli* enzymes are tentatively allocated as type 4 (ReAIV) and type 5 (ReAV) in Class 3. This classification scheme is reflected in Fig. 2 ▸.

## 3. Class 1 asparaginases (formerly known as bacterial-type asparaginases)   

### 3.1. 3D structure of the enzyme and its oligomeric state   

**Figure 3:**
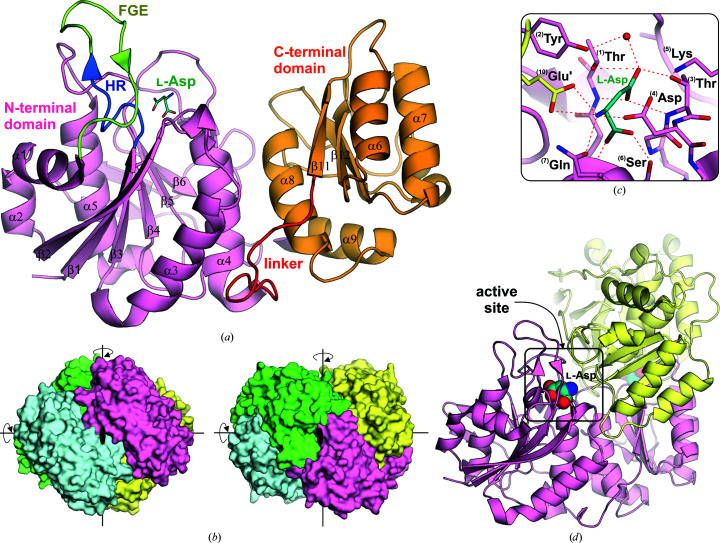
Architecture of bacterial type 2 asparaginases. (*a*) Protomer of EcAII (PDB entry 3eca) with the N-terminal domain in pink, the C-terminal domain in orange, the linker in red, the hinge region (HR) in blue and the flexible gating element (FGE) in green. (*b*) The EcAII homotetramer (a dimer of two intimate dimers) has *D*_2_ (222) symmetry with intimate dimers *A*/*B* (pink/yellow) and *C*/*D* (green/blue). (*c*) Detailed view of the EcAII active site with l-­Asp bound. (*d*) Location of the active site in the *A*/*B* dimer.

To date, in addition to the *E. coli* enzymes, of which EcAII has an especially numerous representation in the PDB, the crystal structures of several other type 2 and type 1 (Class 1) l-asparaginases have been determined (Supplementary Table S1). A typical protomer of type 2 enzymes, for example EcAII, is ∼330 residues long and comprises 14 β-strands and eight α-helices that fold into two distinct, N- and C-terminal, domains (Aghaiypour *et al.*, 2001*b* ▸) connected by an ∼20-residue linker (Fig. 3 ▸). The overall fold of protomers of type 1 (Class 1) asparaginases, for example EcAI, is very similar. The only available structure of a mammalian homolog of bacterial type 1 l-asparaginase is that of the guinea pig enzyme CpAI (PDB entry 4r8k). However, this guinea pig homolog (as well as the human counterpart, HsAI) has an additional ∼200-residue C-terminal domain which probably contains several ankyrin repeats and is not visible in the crystal structure of CpAI (Schalk *et al.*, 2014 ▸). The mammalian enzyme has several other minor changes in comparison to its bacterial counterparts, which include the length and conformation of the linker, the structure of the active-site flexible loop and the presence of an additional loop with a small helix in the N-terminal catalytic domain (Supplementary Fig. S1).

A bacterial type 2 (Class 1) l-asparaginase is a *D*_2_-symmetric homotetramer that can be described as a dimer of two intimate dimers (Swain *et al.*, 1993 ▸; Cerofolini *et al.*, 2019 ▸; Fig. 3 ▸). The bacterial type 1 (Class 1) enzyme EcAI is associated into a similar tetrameric assembly in the crystal packing, but it is not clear whether the protein exists as a tetramer in solution. The same doubt was expressed for the archaeal type 1 enzyme PhAI (from *Pyrococcus horikoshii*), which seems to function as a dimer rather than a tetramer (Yun *et al.*, 2007 ▸). Type 1 asparaginases from some bacterial organisms, for example *Thermus thermophilus*, may act as hexamers (trimers of dimers; Pritsa & Kyriakidis, 2001 ▸), but there is no structural evidence to support this hypothesis.

Sequence comparison of microbial l-asparaginases confirms that intracellular and extracellular enzymes evolved in prokaryotes and eukaryotes independently (Bonthron & Jaskólski, 1997 ▸). A conservative A176V mutation in the intracellular *Saccharomyces cerevisiae* l-asparaginase ScerAI completely abolished the enzyme activity. Sequence and structural comparisons with type 2 bacterial asparaginases show that the mutated residue is in a highly conserved region and plays a vital role in the cohesion of functional tetramers. The fact that type 1 asparaginases show less conservation in this region again suggests that they may have a different quaternary structure while adopting the same subunit fold and intimate dimer architecture.

### 3.2. Allosteric regulation   

**Figure 4:**
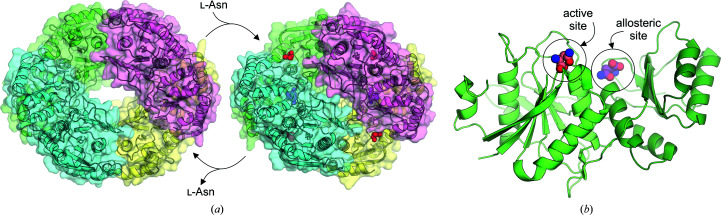
(*a*) Conformational changes accompanying the allosteric regulation of EcAI: left, without l-Asn (PDB entry 2p2d); right, in the presence of l-Asn (PDB entry 2p2n). (*b*) l-Asn located in the active site and allosteric site of the EcAI protomer.

In the crystal structure of EcAI (PDB entry 2p2n), substrate molecules (l-Asn) were found not only in the active site but also in another region of the protein, strongly suggesting that l-Asn can act as an allosteric modulator (Yun *et al.*, 2007 ▸). The EcAI dimer possesses two allosteric sites located close to the dimer interface near Arg240 (Fig. 4 ▸). Signal transmission from the allosteric site to the active site probably involves subtle structural rearrangements at the dimer interface and relocation of Gln118 to facilitate the positioning of the catalytic water molecules (Yun *et al.*, 2007 ▸). EcAI is not the only allosteric asparaginase. The type 1 enzyme from *S. cerevisiae* is also allosteric (Costa *et al.*, 2016 ▸); however, the crystal structure of this yeast enzyme has not been determined to date. On the other hand, not all type 1 enzymes are allosteric. For instance, *P. horikoshii* PhAI might not be allosteric because it seems to permanently be in the catalytically active state (Yun *et al.*, 2007 ▸). Allosteric properties were also not confirmed for *P. furiosus* PfAI, as the quaternary structure of PfAI is almost identical in the ligand-bound and unbound states (Tomar *et al.*, 2014 ▸; Bansal *et al.*, 2012 ▸). The mammalian CpAI enzyme is not allosteric as it follows classic Michaelis–Menten kinetics, while its human counterpart HsAI seems to be allosteric in enzymatic essays, although no crystal structure is available (Schalk *et al.*, 2014 ▸).

### 3.3. Domains and linker   

In Class 1 enzymes, the N-terminal domain has all of the elements necessary for catalysis: the active site with the catalytic threonine residues, a loop acting as a flexible gating element (FGE) and the hinge region (HR) (Fig. 3 ▸). In the bacterial enzymes, the FGE does not comprise well defined secondary-structure elements and in the absence of a substrate it is usually disordered (Papageorgiou *et al.*, 2008 ▸). In contrast, in the mammalian CpAI the FGE is more rigid as it contains a short antiparallel β-sheet formed as a β-hairpin loop (Schalk *et al.*, 2014 ▸; Supplementary Fig. S1). In EcAII, the FGE includes residues 18–31, although some authors also consider the downstream helix to be part of the FGE (Nguyen *et al.*, 2017 ▸). The conserved HR region is formed by a glycine-rich octapeptide: residues 10–17 in EcAII (Lubkowski *et al.*, 2020 ▸).

To build the complete active site, the substrate-binding pocket from one protomer must be complemented by residues from the C-terminal domain of the intimate partner, which are necessary for the stabilization of the substrate in the active site. In contrast to the highly conserved sequences of the N-terminal domains, the sequences of the interdomain linker (Fig. 3 ▸) are highly variable. The length of the linker is correlated with the evolutionary development, and shorter linkers are found in archaeal proteins (Tomar *et al.*, 2014 ▸). As an illustration, the linker in the type 1 asparaginase from *P. horikoshii* (PhAI) is highly strained and seven residues shorter in comparison with that in EcAII (Yao *et al.*, 2005 ▸). Interestingly, the archaeal l-asparaginase from *P. furiosus* (PfAI) obtained from a linker-less recombinant expression of the separate domains was active, indicating that the linker is dispensable for the enzymatic activity (Tomar *et al.*, 2013 ▸).

### 3.4. Conformational states   

The active site of Class 1 asparaginases is located between the subunits of the intimate dimer between strands β1 and β3, which form an unusual left-handed β–α–β crossover (Miller *et al.*, 1993 ▸; Swain *et al.*, 1993 ▸). Each intimate dimer has two complete active sites. The active site can adopt two stable but essentially different conformational states, cat(+) and cat(−), determined by the status of the HR and the FGE and the presence or absence of the substrate (Supplementary Fig. S1). In the absence of the l-Asn substrate, the active site adopts its noncatalytic cat(−) conformation (open conformation, in­active state), with wide-ranging disorder in the FGE region. Upon substrate binding, the FGE changes its conformation to cat(+) (closed conformation, active state), which brings the hydroxyl group of **^(2)^Tyr** 3 towards the substrate and enables the formation of a hydrogen bond between the substrate and **^(1)^Thr** (Supplementary Table S2) from the HR (Lubkowski *et al.*, 2020 ▸). The FGE can prevent the enzyme from binding other ligands before the reaction is completed, so it also acts as ‘post-substrate entry’ gatekeeper.

It seems that the HR and FGE may spontaneously switch conformation between cat(−) and cat(+), but stabilization of the cat(+) state can only be achieved in the presence of a substrate molecule. When a nonhydrolyzable ligand, for example citrate, is bound in the active site, the FGE remains permanently locked, abrogating enzyme turnover (Tomar *et al.*, 2014 ▸). However, it was found that a specific (ordered), rigid β-hairpin structure of the FGE can be present even with an empty active site, for example in the crystal structure of *P. horikoshii* type 1 asparaginase (PhAI), where the FGE with a rigid β-hairpin motif partially covered the active site, limiting substrate access; thus, it can also act as a ‘pre-substrate entry’ gatekeeper (Tomar *et al.*, 2014 ▸; Supplementary Fig. S1). Not only a proper substrate can induce the cat(+) conformation; the same effect is achieved by inorganic ions, for example phosphate (Tomar *et al.*, 2014 ▸; Guo *et al.*, 2017 ▸), sulfate (Lubkowski *et al.*, 2003 ▸) or ammonium ions (Jakob *et al.*, 1997 ▸), that mimic a substrate bound in the active site. However, in some enzymes, proper loop closure is impossible if the ligand in the active site is too large. For instance, l-Gln bound in the active site of ErAII prevents proper conformational transition to the cat(+) state (Nguyen *et al.*, 2016 ▸; Supplementary Fig. S1). Structures of WsAII from *Wolinella succinogenes* revealed that the enzyme may treat the substrate (l-Asn or l-Gln) and the product (l-Asp or l-Glu) in a different way. When l-Asn (or l-Gln) is bound in the active site, a network of hydrogen bonds keeps the FGE in the cat(+) conformation. When the substrate is hydrolyzed to l-Asp (or l-Glu), the hydrogen-bond network is broken to facilitate loop opening and release of the product (Nguyen *et al.*, 2017 ▸). An open state with bound reaction product, representing the terminal catalytic state, has been observed not only in WsAII (Nguyen *et al.*, 2017 ▸) but also in EcAII (Borek *et al.*, 2014 ▸), where such a conformation was interpreted as a crystal-packing artifact.

### 3.5. The catalytic mechanism of Class 1 l-asparaginases   

On the basis of structural and kinetic data, two general catalytic mechanisms have been proposed for Class 1 asparaginases: a single-displacement (or direct-displacement) mechanism was proposed for the mammalian CpAI (Schalk *et al.*, 2016 ▸) and a double-displacement (ping-pong) mechanism was proposed for bacterial enzymes *sensu stricto* (Lubkowski & Wlodawer, 2019 ▸), which is supposed to proceed via a covalent acyl-enzyme intermediate (Palm *et al.*, 1996 ▸). Recent papers unequivocally show that hydrolysis of l-Asn by EcAII follows the double-displacement mechanism, including a sequence of two nucleophilic substitutions. In the ping-pong process, two tetrahedral intermediates (TI1 and TI2) are created in the course of the catalytic cycle. Two important features, namely (i) activation of the nucleophilic threonine by a distant general base (proton sink) and (ii) an unusual structure of the oxyanion hole, including a water molecule, differentiate EcAII, and probably all type 2 and type 1 asparaginases, from other hydrolytic enzymes (Lubkowski *et al.*, 2020 ▸). These studies have identified five residues that are crucial for catalysis by the model Class 1 asparaginase EcAII: Thr12, Tyr25, Thr89, Asp90 and Lys162, with additional residues playing a role in substrate binding and specificity (Lubkowski *et al.*, 2020 ▸). As these residues are conserved among other bacterial enzymes, a unified nomenclature is used in this review: **^(1)^Thr**, **^(2)^Tyr**, **^(3)^Thr**, **^(4)^Asp** and **^(5)^Lys** [Fig. 3 ▸(*c*)]. A summary of the residue numbers in the different enzymes is given in Supplementary Table S2.

**Figure 5:**
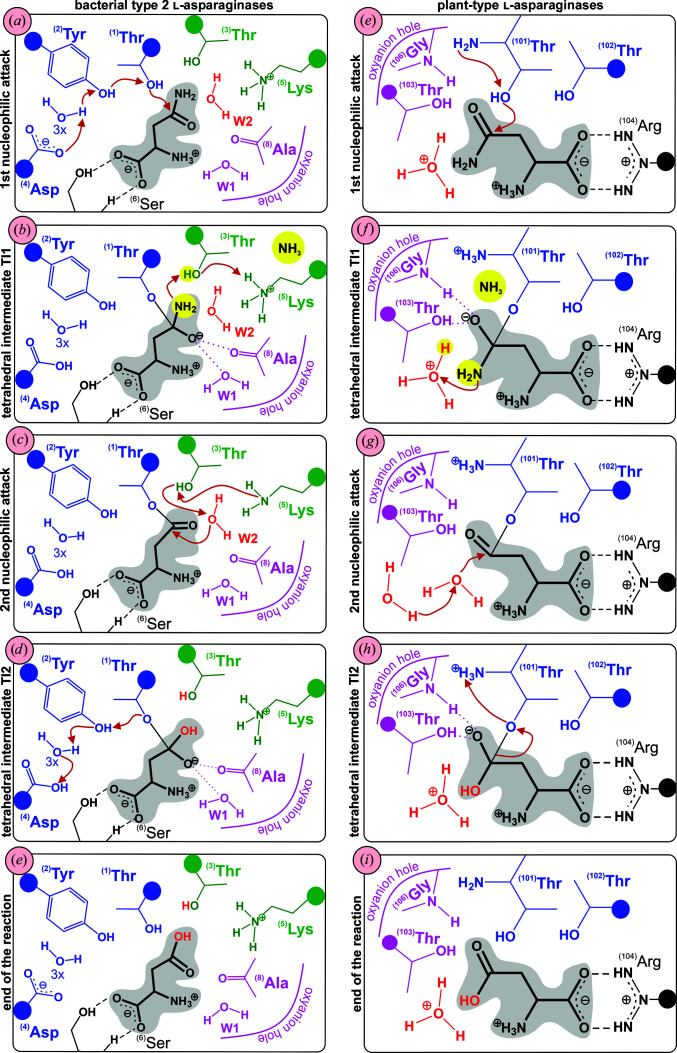
(*a*)–(*e*) Steps in the catalytic mechanism of type 2 bacterial (Class 1) l-asparaginases based on the reactions proposed by Lubkowski *et al.* (2020 ▸). Residues and waters (3×) involved in proton shuttling from **^(1)^Thr** to **^(4)^Asp** via **^(2)^Tyr** are colored blue. **^(3)^Thr** and **^(5)^Lys** that exchange protons with conserved water W2 (red) are colored green. **^(4)^Ala** (carbonyl O atom) and W1 creating the oxyanion hole are colored magenta (the carbonyl O atom of **^(1)^Thr**, which is also part of the oxyanion hole, is not shown). **^(6)^Ser** participating in substrate/product anchoring, the l-Asn substrate, intermediate products and the l-Asp end product are colored black (substrate/product is also hydrogen-bonded to **^(7)^Gln**, **^(8)^Ala**, **^(9)^Asn′** and **^(10)^Glu′**, but these interactions are not shown in the figure). (*f*)–(*j*) Steps of the catalytic mechanism of Class 2 l-asparaginases based on the reactions proposed by Nomme *et al.* (2012 ▸). Protein residues involved in catalysis are colored blue, residues creating the oxyanion hole are colored magenta and water molecules important for catalysis are colored red, **^(104)^Arg** important for substrate anchoring is colored black (substrate/product is also hydrogen-bonded to **^(103)^Thr**, **^(106)^Gly**, **^(105)^Asp** and **^(107)^Gly**, but these interactions are not shown in the figure). In all panels, the directions of the nucleophilic attacks are shown by brown arrows. For clarity, only the functional groups of the protein residues are shown in atomic detail; the remainder is marked by a circle. In all panels, the substrate/product molecules are colored black and shaded.

**Figure 6:**
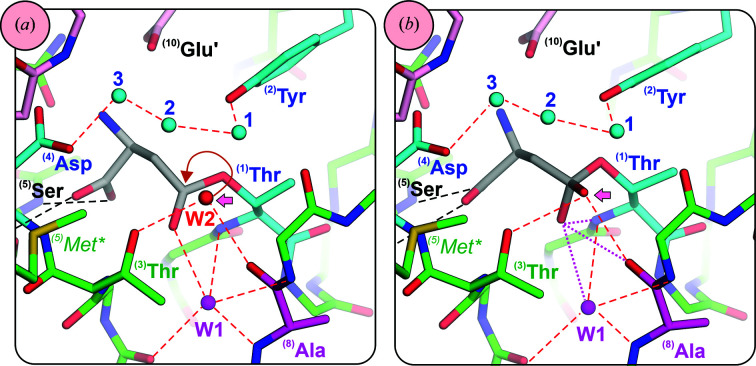
Details of the catalytic mechanism of bacterial (Class 1) type 2 asparaginases. The panels show structures of the K162M mutant of EcAII, in which **^(5)^Lys** was replaced by ^(5)^Met*. (*a*) Acyl-enzyme intermediate (PDB entry 6v2g). (*b*) Tetrahedral intermediate TI2 (PDB entry 6v25). Residues and waters (W1, W2, W3) participating in proton shuttling from **^(1)^Thr** to **^(4)^Asp** via **^(2)^Tyr** are colored cyan. **^(3)^Thr** and ^(5)^Met* (corresponding to **^(5)^Lys** in the wild-type protein) are colored green. Red dashed lines represent hydrogen bonds between waters and residues involved in catalysis. Magenta dotted lines show the interactions stabilizing the TI2 intermediate in the oxyanion hole formed by the carbonyl O atom of **^(8)^Ala** and the conserved water molecule W1. A fat pink arrow marks the position of water W2 in (*a*), which is incorporated into TI2 in (*b*). Black dashed lines represent hydrogen bonds anchoring the substrate/product in the active site. Some less important hydrogen bonds were omitted for clarity. A brown curved arrow indicates the direction of the nucleophilic attack of water W2 on the acyl-enzyme intermediate.

In a bacterial type 2 enzyme such as EcAII, initiation of the catalytic process requires precise orientation of the substrate molecule, completed by the conformational change of the HR and transition of the FGE from the cat(−) to the cat(+) state. The conformational changes are driven by electrostatic forces and lead to the polarization of the **^(1)^Thr** hydroxyl group (Lubkowski *et al.*, 2020 ▸). **^(1)^Thr** acts as a weak nucleophile without the direct assistance of a general base which could enhance its nucleophilic properties. The role of general base can be attributed to the carboxylate of **^(4)^Asp**; however, this residue is distant from **^(1)^Thr**. The first nucleophilic attack is launched by **^(1)^Thr** and the reaction proceeds by covalent bond formation between **^(1)^Thr** (O^γ^) and the C^γ^ atom of the substrate [Fig. 5 ▸(*a*)]. The proton abstracted from the nucleophile is transferred through **^(2)^Tyr**, which acts as a proton conveyor, and a network of water molecules to **^(4)^Asp**. The α-carboxylate group of the substrate and the negatively charged side chain of **^(4)^Asp** form a proton sink which increases the efficiency of the first nucleophilic attack, leading to the formation of the negatively charged tetrahedral intermediate TI1 (Fig. 6 ▸), stabilized by the oxyanion hole [Fig. 5 ▸(*b*)]. The oxyanion hole is formed by the backbone amides of **^(1)^Thr** and **^(8)^Ala**, and by a conserved water molecule W1 (Figs. 5 ▸ and 6 ▸). Next, the molecule of ammonia is liberated from the substrate, which remains covalently attached to **^(1)^Thr** [Fig. 5 ▸(*b*)].

The release of NH_3_ initiates the second displacement step. The leaving –NH_2_ group of the substrate accepts a proton from the OH group of **^(3)^Thr**, which in turn accepts one from **^(5)^Lys**. After the release of ammonia, **^(5)^Lys** remains uncharged [Fig. 5 ▸(*c*)] and recovers protonation from water W2 [Fig. 5 ▸(*c*)], which like water W1 is conserved in all type 2 l-asparaginases. The shuttle of protons activates water molecule W2 (Fig. 6 ▸), making it a strong nucleophile (OH^−^) to start the second displacement reaction, which leads to the second tetrahedral intermediate TI2 [Fig. 5 ▸(*d*)]. The proton from the carboxylic group of **^(4)^Asp** is transferred back to **^(1)^Thr** via the same network of water molecules and **^(2)^Tyr**. The covalent bond between **^(1)^Thr** and the product becomes weaker and finally breaks, leading to the formation of the l-Asp product [Fig. 5 ▸(*e*)]. The departure of l-Asp recreates the original state of the active site: the HR and FGE return to the cat(−) conformations and the enzyme becomes ready for the next catalytic cycle. In EcAII, the rate of the net enzymatic reaction is controlled by the rate of the first displacement step (Lubkowski *et al.*, 2020 ▸). This step is irreversible due to the dissociation of NH_3_ from the active site, while the second nucleophilic substitution, carried out by a water molecule, is fully reversible.

It is important to note that positioning of the catalytic residues in Class 1 type 1 enzymes differs from that in type 2, especially for tyrosine **^(2)^Tyr**. In type 1 enzymes, **^(2)^Tyr′** comes from the complementary subunit (denoted by ′) of the intimate dimer (Supplementary Fig. S2). The type 1 enzymes also lack a **^(10)^Glu′** counterpart (Supplementary Table S2). These arrangements of residues also occur in the mammalian enzyme CpAI, where **^(2)^Tyr′** closing the active site in the cat(+) state is likewise located in a C-terminal loop of the complementary protomer, not in the FGE [Supplementary Fig. S2(*e*)]. Upon ligand binding this C-terminal loop becomes more ordered and the new conformation locates **^(2)^Tyr′** in close vicinity of the catalytic **^(1)^Thr** residue (Schalk *et al.*, 2014 ▸).

Interestingly, careful analysis of the sequences and crystal structures has allowed the detection of possible errors in asparaginase classification. For example, the enzyme from *Campylobacter jejuni* (PDB entry 3nxk; Center for Structural Genomics of Infectious Diseases, unpublished work) was assigned in UniProt as the product of the *ansA* gene, while the architecture of the active site strongly suggests that it belongs to the bacterial type 2 enzymes (Supplementary Table S2). The same mistake was made for *Helicobacter pylori* aspara­ginase (UniProt gene *ansA*), where the active-site arrangement and substrate affinity suggest that it is a type 2 enzyme (PDB entry 2wlt; Dhavala & Papageorgiou, 2009 ▸). As there are different origins of **^(2)^Tyr**/**^(2)^Tyr′** in type 2/type 1 aspara­ginases, the structure of the enzyme in the cat(+) state can be treated as an important criterion (together with substrate affinity and sequence similarity) of bacterial asparaginase classification.

The positioning of **^(2)^Tyr** close to **^(1)^Thr** is necessary to initiate the catalytic process. However, this event will have slightly different consequences in type 2 and type 1 enzymes. In type 1 enzymes, **^(2)^Tyr′** is hydrogen-bonded only to **^(1)^Thr** [Supplementary Figs. S2(*b*)–S2(*d*)], while in type 2 enzymes it is also hydrogen-bonded to a chain of three conserved water molecules [Supplementary Fig. S2(*a*)]. In this context, the scheme of the catalytic reaction described for EcAII is probably true for the whole family of microbial type 2 l-asparaginases (Lubkowski *et al.*, 2020 ▸) and glutamin­ase-asparaginases (G-As) (Supplementary Fig. S3). However, docking of l-Gln in a G-A enzyme seems to proceed via two steps and is more complex than in the case of l-Asn (Strzelczyk *et al.*, 2020 ▸). The probable difference in the catalytic mechanisms of Class 1 type 2 and type 1 asparaginases is related to the mode of proton transfer between **^(1)^Thr** and **^(4)^Asp**, but this mechanism has not been clarified to date. Interestingly, the structural analysis of the mammalian CpAI and its mutants unambiguously revealed that the substrate does not form a covalent acyl-enzyme intermediate with **^(1)^Thr**, in contrast to the scenario described for EcAII. This observation argues in favor of the direct-displacement mechanism in the mammalian enzymes. Another piece of evidence is the absence of so-called ‘burst kinetics’ in CpAI, which is typical for enzymes utilizing the ping-pong mechanism (Schalk *et al.*, 2016 ▸). An excellent review of the mechanism of Class 1 asparaginases has been recently published by Lubkowski & Wlodawer (2021 ▸).

### 3.6. Substrate specificity and inhibitors of Class 1 asparaginases   

Another classification of asparaginases is based on enzymatic activity and substrate specificity. In this scheme, Class 1 l-amidohydrolases can be divided into two major groups. The first includes enzymes that primarily utilize l-asparagine as their substrate and have low or very low l-glutaminase activity (for example EcAII and ErAII). The second group includes enzymes, referred to as glutaminase-asparaginases, which hydrolyze l-Asn and l-Gln with a similar efficiency [for example *Pseudomonas* 7A glutaminase-asparaginase (PGA) and *Acinetobacter glutaminasificans* glutaminase-asparaginase] (Aghaiypour *et al.*, 2001*b* ▸; Jakob *et al.*, 1997 ▸).

In addition to l-Asn, bacterial type 1 and type 2 aspara­ginases can also accept other substrates, such as l-Gln, d-Asn,^4^ and succinic monoamide (Supplementary Fig. S4). Structures of complexes of ErAII with l-Glu, d-Asp and succinic acid (Suc) revealed a similar binding mode for all of these ligands. These compounds are products of the enzymatic reaction; however, they also can serve as substrates when protonated (Aghaiypour *et al.*, 2001*b* ▸). Well known irreversible inhibitors of asparaginases (and glutaminases) can be found among diazo derivatives of amino acids, *e.g.* l-DON (6-diazo-5-oxo-l-norleucine) and l-DONV (5-diazo-4-oxo-l-norvaline), but inhibitory activity is also attributed to citrate ions (Fig. S4) (Lubkowski *et al.*, 2019 ▸). However, the chemistry of the reactions of the enzymes with diazo inhibitors might be significantly different from their mechanisms with natural substrates because of the high reactivity of diazo compounds (Aghaiypour *et al.*, 2001*a* ▸). The crystal structures revealed that the diazo inhibitors are covalently bound to the nucleophilic **^(1)^Thr** but also to **^(2)^Tyr** from the FGE [Supplementary Fig. S3(*b*)], suggesting that the mode of inhibition is different from that of citrate ion, which only reacts with **^(1)^Thr** [Supplementary Fig. S3(*c*)]. The crystal structures of PGA inhibited with l-DON and l-DONV showed that the covalent linkage to **^(1)^Thr** is critical for the inhibition process, while the covalent linkage to **^(2)^Tyr** permanently locks the FGE in the closed conformation (Ortlund *et al.*, 2000 ▸). Similar observations were made for ErAII complexed with l-DON (or d-DON). Both inhibitors were covalently bound to **^(1)^Thr** and **^(2)^Tyr**, but they did not affect the conformations of residues in the active site or the positions of the conserved water molecules (Aghaiypour *et al.*, 2001*a* ▸).

Some residues located in the active site play a role in defining substrate specificity. The natural substrate, l-Asn, fits perfectly to the active site of type 2 and type 1 asparaginases. It has also been shown that an alternative mode of l-Asn orientation is possible in EcAI (Yun *et al.*, 2007 ▸). Binding the larger l-Gln requires more sophisticated gymnastics within the active site of bacterial asparaginases (Nguyen *et al.*, 2016 ▸). The specificity of l-asparaginases towards l-Asn or l-Gln seems to be determined by two residues in the close vicinity of the active site, positions **^(7)^Gln** and **^(9)^Asn′** (Supplementary Table S2; Aghaiypour *et al.*, 2001*b* ▸; Yun *et al.*, 2007 ▸). Similarly to EcAII, the *H. pylori* l-asparaginase HpAII, with negligible l-glutaminase activity, has a triad, **^(7)^Gln**, ^(**9)**^ **Asn′** and **^(10)^Glu′**, near the catalytic region that is typical of enzymes with dominating l-asparaginase activity. These residues modulate the accessibility of the active site, excluding larger substrates such as l-Gln. Enzymes with significant glutaminase co-activity, as in all G-A enzymes, have Glu and Ser at positions corresponding to **^(7)^Gln** and **^(9)^Asn′**, respectively (Supplementary Fig. S3 and Table S2). Other substitutions (Supplementary Table S2) at positions **^(7)^Gln**/**^(9)^Asn′** in bacterial type 2 enzymes include **^(7)^Gln**/**^(9)^Ser′** in ErAII and EwAII (both have a relatively low glutaminase co-activity; Nguyen *et al.*, 2016 ▸; Kravchenko *et al.*, 2008 ▸) and **^(7)^Ser**/**^(9)^Ser′** in CjAII (no kinetic data). In type 1 enzymes other combinations are present: **^(7)^Ser**/**^(9)^Asn′** in EcAI (no detectable glutaminase activity; Yun *et al.*, 2007 ▸), YpAI and VcAI (no kinetic data) and **^(7)^Thr** **/^(9)^Gly′** in PfAI, PhAI and TkAI (all have negligible glutaminase activity; Saeed *et al.*, 2020 ▸; Bansal *et al.*, 2010 ▸; Chohan *et al.*, 2020 ▸).

Another residue affecting glutaminase co-activity is proline located in close proximity to the catalytic **^(1)^Thr**. In *H. pylori* HpAII **^(1)^Thr** is flanked by Asn123 instead of the proline present at the corresponding position of EcAII. This change reduces the hydrophobicity around **^(1)^Thr** and may have an additional influence on the substrate preferences of HpAII, which has negligible glutaminase activity (Dhavala & Papageorgiou, 2009 ▸). On the other hand, WsAII from *W. succinogenes* has two variants with Pro/Ser121 flanking **^(1)^Thr**. It was found that the proline variant has l-glutaminase activity, while the serine variant is free of glutaminase activity. Crystallographic studies revealed that residue 121 affects the conformation of the conserved **^(2)^Tyr** of FGE. When l-Gln is docked in the active site, steric clashes with the extra methylene group prevent loop closure in the Ser121 variant. In contrast, the circular side chain of Pro121 participates in CH⋯π aromatic interactions with **^(2)^Tyr**, stabilizing the cat(+) state even in the presence of l-Gln (Nguyen *et al.*, 2017 ▸).

## 4. Class 2 asparaginases (formerly known as plant-type asparaginases)   

### 4.1. Architecture of Class 2 l-asparaginases   

Plant-type (type 3, Class 2) asparaginases are present not only in plants but also in microorganisms, insects and mammals, including humans (Borek & Jaskólski, 2001 ▸). They belong to the Ntn-hydrolase family (Brannigan *et al.*, 1995 ▸) and usually have dual isoaspartyl aminopeptidase/l-asparaginase activity (EC 3.5.1.1/3.4.19.5). Ntn-hydrolases are expressed as inactive precursors that develop enzymatic activity upon autoproteolytic maturation. The autoproteolysis involves either the removal of a pro-peptide or cleavage of the precursor chain (Michalska *et al.*, 2008 ▸). The mechanism of autocleavage is diversified between different Ntn-enzymes and also among Class 2 asparaginases (Michalska *et al.*, 2008 ▸; Nomme *et al.*, 2014 ▸).

**Figure 7:**
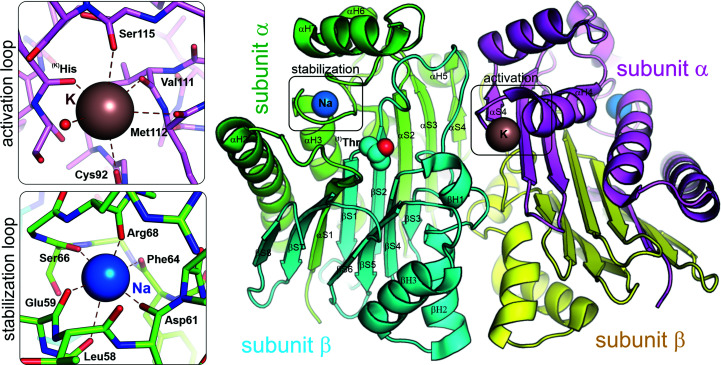
Crystal structure of the mature form of the Class 2 potassium-dependent l-asparaginase from *P. vulgaris* (PDB entry 4pv2). The mature protein is a heterotetramer built of two α subunits (green and magenta) and two β subunits (cyan and yellow). All Class 2 l-asparaginases possess a stabilization loop with a coordinated Na^+^ ion (blue sphere). Potassium-dependent enzymes also possess an activation loop that is capable of coordinating a K^+^ ion (brown sphere).

The most thoroughly studied Class 2 l-asparaginases are human HsAIII (Nomme *et al.*, 2014 ▸, 2012 ▸; Su *et al.*, 2013 ▸) and *E. coli* EcAIII (Michalska *et al.*, 2005 ▸, 2008 ▸; Borek & Jaskólski, 2000 ▸). These proteins come from very distant taxonomic groups but share high structural similarity (r.m.s.d. of 0.6 Å). This structural homology is also shared by other Class 2 enzymes. However, except for EcAIII, HsAIII and their mutants, the PDB only contains structures of the enzymes from yellow lupin (*L. luteus*; LlAIII; Michalska *et al.*, 2006 ▸), kidney bean (*P. vulgaris*; PvAIII; Bejger *et al.*, 2014 ▸) and guinea pig (*Cavia porcellus*; CpAIII; Schalk & Lavie, 2014 ▸), and all of these structures are almost identical (r.m.s.d. of 0.6–0.8 Å; Supplementary Table S3). All Class 2 asparaginases have the same architecture arising from two copies of a 33–35 kDa precursor assembled into an inactive dimer (Supplementary Fig. S5). During the maturation process, each precursor is autocatalytically cleaved into the N-terminal subunit α (∼20 kDa) and C-terminal subunit β (∼15 kDa). After maturation, the whole assembly can be viewed as a heterotetramer or as a dimer of two αβ heterodimers. Structurally, the heterodimer has αββα^5^ sandwich topology, with the core composed of two β-sheets flanked on each side by a layer of α-helices. The smaller β-sheet consists of four antiparallel β-strands belonging to chain β, while the larger sheet has mostly antiparallel character and is made of eight strands contributed by both chains (Fig. 7 ▸).

Class 2 asparaginases share common structural elements that are crucial for their enzymatic performance: a sandwich-like fold of two distinct subunits connected by a linker (Supplementary Fig. S5), an autoproteolytic and catalytic center with a conserved threonine triad and other conserved elements, the His-Gly-Gly (HGG) loop and binding site for autoprocessing accelerators, and two alkali-metal binding loops: the stabilization loop and activation loop (Fig. 7 ▸). In the following description of the structure and catalytic mechanisms, similarly to the system adopted in Section 2, a unified residue numbering will be used, with the corresponding numbers in each particular enzyme listed in Supplementary Table S4.

Chains α and β of Class 2 enzymes, forming the two structural domains of the precursor protein, are connected via a linker or variable loop. The linker has a highly variable sequence and is usually not visible in crystal structures (Supplementary Fig. S5), which suggests its high dynamic disorder or its processive C-terminal degradation after the incision in front of the catalytic **^(101)^Thr** (Michalska *et al.*, 2008 ▸). However, in the structure of the EcAIII T179A mutant (with the catalytic threonine replaced by an alanine) it was possible to model as many as 11 residues upstream of the **^(100)^Gly-^(101)^Thr** cleavage point (PDB entry 3c17; Michalska *et al.*, 2008 ▸). The visible part of the linker in this structure tightly covers access to the active site (Supplementary Fig. S5). This might suggest that the linker protects the immature protein from degradation by other proteases or premature autoactivation under suboptimal conditions. Interestingly, in the crystal structure of HsAIII in the pre-cleaved state co-crystallized with glycine (which was used as an autoprocessing activator; see Section 4.2), it was possible to model a large fragment of the linker (PDB entry 4osx). Its conformation is different from that in uncleaved EcAIII; however, this might be due to the presence of the glycine molecule close to the scissile **^(100)^Gly**-**^(101)^Thr** bond and its interactions with the linker residues (Supplementary Fig. S6). This suggests that the glycine molecule not only activates the nucleophilic **^(101)^Thr** but possibly also affects the conformation of the linker itself, helping to ‘uncover’ the active site, thus facilitating the autoactivation process.

### 4.2. Autoproteolytic maturation   

The funnel-shaped active site of Class 2 asparaginases is located between the two β-sheets of the αβ heterodimer, with an entrance surrounded by loops from both subunits (Michalska *et al.*, 2005 ▸). The active site is formed as a conserved threonine triad **^(101)^Thr**-**^(102)^Thr**-**^(103)^Thr** (Supplementary Table S4). This site has dual activity: it hydrolyzes its asparagine (and isoaspartyl dipeptide) substrates, and it is also responsible for the cleavage of the inactive precursor in the self-maturation process. The precursor is split into subunits α and β to liberate the catalytic **^(101)^Thr** at the N-terminus of subunit β (Michalska *et al.*, 2008 ▸; Nomme *et al.*, 2012 ▸). A series of mutants were generated to elucidate the role of the threonine triad in autoprocessing (Nomme *et al.*, 2014 ▸). It was established that correct positioning of the **^(101)^Thr** hydroxyl group is critical for autocleavage, while **^(102)^Thr** and **^(103)^Thr** are not essential for this process. It seems that a torsional strain at the **^(101)^Thr** side chain in the immature precursor is the driving force of the autoprocessing reaction (Li *et al.*, 2012 ▸). The cleavage relieves the torsional restriction of **^(101)^Thr** and this process is facilitated by a conformational change of the HGG loop [Supplementary Fig. S6(*b*)], which is conserved in Class 2 asparaginases (Nomme *et al.*, 2014 ▸). It is also possible that a conformational flip of the first glycine residue may promote structural changes that lead to the initiation of the autocleavage reaction (Nomme *et al.*, 2012 ▸). Substitution of the glycine residues in the HGG loop by alanines resulted in a sharp decrease in the autoprocessing rate. It was found that the cleaved and uncleaved states may also differ in the position of the whole fragment harboring the HGG element (Li *et al.*, 2012 ▸).

**Figure 8:**
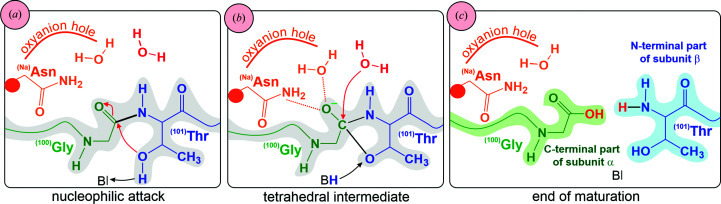
(*a*)–(*c*) Schematic representation of the mechanism of maturation of Class 2 asparaginases. The process is started by the nucleophilic attack of **^(101)^Thr** on the scissile bond **^(100)^Gly**-**^(101)^Thr** (black). The reaction needs a water molecule (red) and assistance of the general base **B**. The oxyanion hole (orange) is made by **^(Na)^Asn** from the stabilization loop and another water molecule (orange). At the end of the maturation process, the protomer is cleaved into two subunits: α (green) and β (blue).

**Figure 9:**
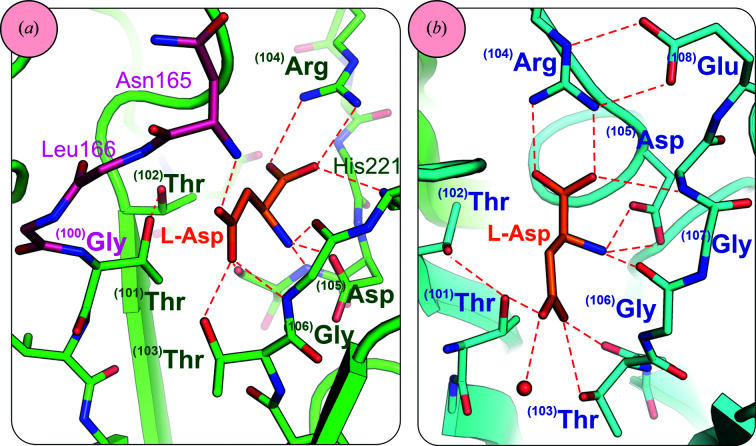
Details of l-Asp (orange) binding in Class 2 asparaginases. (*a*) l-Asp bound in the active site of immature HsAIII (PDB entry 4pvr); a fragment of the linker region is shown in dark pink. (*b*) l-­Asp in the active site of mature ECAIII and the residues stabilizing its binding (PDB entry 2zal).

The autocleavage starts with a nucleophilic attack of the side chain of **^(101)^Thr** on the carbonyl C atom of the peptide bond in front of it (Li *et al.*, 2012 ▸; Fig. 8 ▸). **^(101)^Thr** is involved in a network of hydrogen bonds that make its OH proton labile. The most important interaction is the hydrogen bond to the hydroxyl group of **^(102)^Thr**, the position of which is stabilized by hydrogen bonds [Supplementary Fig. S6(*a*)] to a conserved tandem of glycine residues in front of it. In the next step, a tetrahedral transition state is built and stabilized by the oxyanion hole formed by **^(Na)^Asn** from the sodium-binding loop and a water molecule. This oxyanion hole is different from that utilized by the mature enzyme for the asparaginase reaction. In the final step, a nucleophilic water molecule hydrolyzes the tetrahedral transition state to complete the reaction (Michalska *et al.*, 2008 ▸). The self-maturation process not only liberates the catalytic **^(101)^Thr** but also provides the environment for substrate binding. The crystal structure of l-Asp in complex with uncleaved HsAIII revealed that in the immature protein the substrate (Fig. 9 ▸) cannot be correctly oriented with respect to **^(101)^Thr** and therefore no hydrolysis can take place (Nomme *et al.*, 2012 ▸).

Several studies have indicated that different Class 2 asparaginases have different autoproteolysis rates and that in some cases the process can be accelerated by small-molecule activators, at least in the case of human and guinea pig asparaginases (Schalk & Lavie, 2014 ▸). The first such activator to be reported was glycine (Su *et al.*, 2013 ▸), which accelerates autoprocessing *in vitro* and *in vivo* (Li *et al.*, 2016 ▸). Crystal structures of HsAIII–Gly complexes show that two glycine molecules can bind to the enzyme in the pre-cleaved state [Supplementary Fig. S6(*c*)]. One mimics the l-Asn substrate in the active site and is hydrogen-bonded to the conserved **^(104)^Arg** residue, while the other is positioned near the scissile **^(100)^Gly**-**^(101)^Thr** bond (Su *et al.*, 2013 ▸). It is possible that in the interaction with **^(101)^Thr** the glycine activator acts as a base for proton abstraction from the hydroxyl group, activating its nucleophilic character and promoting its attack on the preceding peptide bond.

Amino acids similar to glycine, such as alanine or serine, seem to be too large to fit the binding site near the scissile **^(100)^Gly**-**^(101)^Thr** bond and do not accelerate autoproteolysis (Su *et al.*, 2013 ▸). It has been suggested that the glycine activator is first bound to **^(104)^Arg** and only later near the scissile bond, as the **^(104)^Arg/Gln** mutant (R196Q in HsAIII) requires a very high glycine concentration to rescue the autoprocessing, while without glycine no autoprocessing of the HsAIII R196Q variant takes place (Li *et al.*, 2016 ▸). Tests of other compounds revealed that sarcosine, carbamate and phosphate also promoted the cleavage, albeit much less efficiently. Sarcosine, for instance, accelerated the cleavage, while its isostere β-alanine did not, indicating that the position of the glycine amino group provides the interactions necessary for the cleavage reaction. Ammonium, β-alanine, betaine, O-phosphorylethanolamine, glycinamide, bicarbonate, taurine and l-asparagine (the substrate) do not accelerate the autoproteolysis reaction (Li *et al.*, 2016 ▸).

A recent study has revealed that the autoprocessing of Class 2 asparaginases depends on the oligomeric state (Li *et al.*, 2016 ▸). In the studies reported for HsAIII, measurements of *T*_m_ as a function of time during autoproteolysis allowed three different molecular species to be distinguished: αβ|βα (unprocessed homodimer), α/β|βα (half-processed heterotrimer) and α/β|β/α (fully processed heterotetramer). Auto­processing removes the structural strain accumulated at **^(101)^Thr** in the uncleaved protein and increases the thermal stability (melting temperature *T*_m_) from 61°C for αβ|βα to 70°C for the fully cleaved α/β|β/α species, while the half-processed α/β|βα form has a *T*_m_ of ∼65°C. The same study revealed that the cleavage rate of the first αβ protomer is significantly faster than the cleavage of the second protomer (Li *et al.*, 2016 ▸). It is thus possible that cleavage of the first protomer results in allosteric rearrangements at the dimer interface that may reduce the torsional strain at **^(101)^Thr** of the second protomer or shift the equilibrium of the unprocessed active site away from the cleavable state. Also, cleavage of the first protomer may result in an entropy increase that could lower the probability of a strained conformation in the second protomer (Li *et al.*, 2016 ▸). It was also suggested that cleavage of the second protomer is supported by the association of an additional monomer, leading to the formation of a transient trimeric state. The trimerization would trigger a conformational switch in the half-processed dimer, rebuilding the torsional strain in the second protomer (Morais *et al.*, 2020 ▸). However, this hypothesis needs to be verified by additional studies, as to date there are no other reports to suggest the possibility of HsAIII trimerization.

Site-directed mutagenesis at the dimer interface of HsAIII showed that dimer formation is also critical for autoproteolysis (Li *et al.*, 2016 ▸). In mutants such as C202A or C202S the dimerization process was weaker but autoprocessing recovery was possible in the presence of an activator (glycine), and the fully cleaved variants had nearly wild-type kinetics of substrate hydrolysis. It was thus concluded that l-asparaginase activity and autoprocessing are separate molecular events and are unrelated (Li *et al.*, 2016 ▸), even if carried out by the same catalytic center.

### 4.3. Substrate binding and architecture of the binding site   

The dominant enzymatic activity of Class 2 asparaginases is attributed to the hydrolytic decomposition of isoaspartyl (β-aspartyl) dipeptides, while their affinity for l-asparagine is relatively weak (millimolar range; Michalska *et al.*, 2005 ▸; Cantor *et al.*, 2009 ▸). Isoaspartyl residues are formed spontaneously at l-Asn sites upon protein aging. They introduce unnatural kinks in the protein chain, disrupting normal folding and leading to toxic effects (Aswad *et al.*, 2000 ▸). Proteins with isoaspartyl residues are repaired or degraded by proteases. The latter process produces β-peptides, which are further hydrolyzed by specialized isoaspartyl peptidases. The enzymatic degradation of β-aspartyl dipeptides by Class 2 asparaginases has never been investigated in detail, so the catalytic mechanism is not known. On the other hand, the mechanism of l-Asn hydrolysis has been intensively studied for ∼20 years due to the potential application of the plant-type enzymes in leukemia therapy (Nomme *et al.*, 2012 ▸; Schalk & Lavie, 2014 ▸; Michalska *et al.*, 2005 ▸, 2006 ▸).

Two of the conserved threonines in the **^(101)^Thr**-**^(102)^Thr**-**^(103)^Thr** triad are connected by a network of hydrogen bonds [Supplementary Fig. S6(*a*)]. The same arrangement of hydrogen bonds exists in the free enzyme and in enzyme–product complexes, so the binding of a substrate/product in the active site does not seem to affect the conformation of the catalytic residues. **^(101)^Thr** acts as the Ntn-nucleophile, *i.e.* is the N-terminal residue of subunit β (β-strand βS1; Fig. 7 ▸), **^(102)^Thr** is located in β-strand βS2 and **^(103)^Thr** is positioned at β-strand βS3. In the mature enzyme, the nucleophilic **^(101)^Thr** is the acceptor of a hydrogen bond from the **^(102)^Thr** OH group, while **^(102)^Thr** is an acceptor of two N–H⋯O hydrogen bonds from the neighboring glycine residues that secure the proper conformation of **^(102)^Thr** (Michalska *et al.*, 2008 ▸) [Supplementary Fig. S6(*a*)]. The catalytic **^(101)^Thr** and other conserved residues, **^(103)^Thr**, **^(104)^Arg**, **^(105)^Asp**, **^(106)^Gly** and **^(107)^Gly** (Supplementary Table S4), located in the β subunit are involved in substrate (product) binding [Fig. 9 ▸(*b*)]. Residue **^(104)^Arg** forms a salt bridge with the α-carboxylate group of the substrate/product. This interaction is responsible for the anchoring of the substrate/product in the active site and explains the specificity of the enzyme, which is limited to substrates with a free α-carboxyl group. The side chain of **^(104)^Arg** is held in place by **^(108)^Glu**. **^(107)^Gly** forms a hydrogen bond to one of the O atoms of the α-carboxyl group, while **^(105)^Asp** and **^(106)^Gly** are responsible for anchoring the α-amino group of the substrate/product [Fig. 9 ▸(*b*)]. Residues **^(103)^Thr** and **^(106)^Gly**, via their N–H groups, form the oxyanion hole that stabilizes the negative charge developing on the substrate β carbonyl group in the tetrahedral transition state (Michalska *et al.*, 2005 ▸).

### 4.4. Catalytic mechanism   

The **^(101)^Thr** nucleophile is the catalytic residue poised for a ping-pong mechanism of l-Asn hydrolysis, which, despite all of the structural differences, suggests a mechanistic similarity to Class 1 asparaginases. The structural differences include a fundamentally different quaternary structure of Class 2 and Class 1 enzymes, but the situation in the active site looks surprisingly similar: there are also two threonine residues, **^(101)^Thr** and **^(103)^Thr**, which could perform a ping-pong mechanism, and likewise there is a (geometrically) problematic proton abstractor (the N-terminus of subunit β) that is supposed to activate the primary nucleophile of **^(101)^Thr**. The identity of the final water nucleophile also needs confirmation.

The hydrolysis of l-asparagine is dependent on the free amino group of **^(101)^Thr**, which in Ntn-hydrolases is supposed to activate its hydroxyl group, while conserved water molecules located in the close vicinity (and held in position by the adjacent residues from the sodium-binding loop) aid in proton transfer. The function of these water molecules is to stabilize the protonated amino group once it has abstracted the proton from the **^(101)^Thr** hydroxyl. The mechanism of l-Asn hydrolysis proposed by Michalska *et al.* (2005 ▸) was expanded by Nomme *et al.* (2012 ▸). It can be described as a sequence of two nucleophilic substitutions [Figs. 5 ▸(*f*)–5 ▸(*j*)].

The reaction starts with the nucleophilic attack of **^(101)^Thr** on the substrate and this initiates the first displacement step. The negatively charged tetrahedral transition state at the C^γ^ atom of the substrate is stabilized by the oxyanion hole formed by **^(103)^Thr** and **^(106)^Gly** [Figs. 5 ▸(*f*)–5 ▸(*j*)] (Michalska *et al.*, 2005 ▸; Nomme *et al.*, 2012 ▸). The covalent transition state also affects the conformation of the conserved HGG loop, suggesting that the HGG motif is involved not only in autoprocessing but also in the l-asparaginase activity. Collapse of the tetrahedral transition state releases a molecule of ammonia and leaves the substrate covalently attached (via β-acylation) to **^(101)^Thr**. A water molecule coordinated in the close vicinity of the β-acyl linkage probably acts as a proton donor for the leaving –NH_2_ group. The release of NH_3_ initiates the second displacement event. The attack of a water molecule on the covalent intermediate results in the formation of another tetrahedral transition state and the reaction is terminated by release of the l-Asp product and the return of the enzyme to the original state (Nomme *et al.*, 2012 ▸).

### 4.5. Regulation of the enzymatic activity: metal-binding loops and catalytic switch   

Plant-type asparaginases have a conserved structural element referred to as the sodium-binding loop (or stabilization loop) located near the active site in the α chain. The stabilization loop creates a positional lock on the catalytic **^(101)^Thr** residue by forming a hydrogen bond between its free amino group and the adjacent **^(Na)^Asn** residue (Fig. 7 ▸). This interaction is necessary for proper orientation of **^(101)^Thr**. The conformation of the stabilization loop is maintained by a sodium cation with an octahedral coordination sphere formed by six carbonyl groups from the main chain within the stabilization loop sequence (Michalska & Jaskolski, 2006 ▸).

Depending on their activity profiles, Class 2 l-asparaginases can be further divided into potassium-dependent and potassium-independent enzymes (Fig. 2 ▸; Sodek *et al.*, 1980 ▸; Bejger *et al.*, 2014 ▸). Potassium-dependent plant-type asparaginases possess yet another loop, which acts to control the enzymatic activity (Bejger *et al.*, 2014 ▸). This activation loop can switch the enzyme between ON/OFF states in the presence/absence of potassium cations (Supplementary Fig. S7). When K^+^ is coordinated in the activation loop, the side chain of **^(104)^Arg**, which is responsible for substrate binding, has a conformation that enables anchoring of the substrate/product in the active site (ON state). When potassium is not bound or is replaced by Na^+^, the loop undergoes a rearrangement that switches the enzyme to the OFF state (Supplementary Fig. S7). This conformational change has a dramatic effect on three side chains forming a ‘catalytic switch’: **^(K)^His**-**^(K)^Arg**-**^(K)^Glu** (Supplementary Table S4). During this transformation, the side chain of **^(K)^His** moves into the protein core, taking up the space usually occupied by the side chain of **^(104)^Arg**. To avoid steric clashes, the side chain of **^(104)^Arg** rotates by 180° and in this new position is no longer able to bind to the substrate/product in the active site (OFF state). The movement of **^(104)^Arg** to the OFF state triggers a rotation of the **^(108)^Glu** side chain, which no longer stabilizes **^(104)^Arg** in the ON state (Bejger *et al.*, 2014 ▸). All of these changes also affect the position of the whole helix at the C-terminal fragment of chain α and of the neighboring loops. The catalytic switch can be described as an allosteric process, as coordination of K^+^ in the activation loop of one protomer affects the position of **^(104)^Arg** in the second protomer.

In potassium-independent asparaginases, the structural element that could be viewed as the activation loop also exists; however, it is unable to bind metal ions. It was confirmed that the **^(K)^Ser** residue is crucial for metal coordination in the potassium-dependent enzymes (Supplementary Table S4). If **^(K)^Ser** is replaced by isoleucine, the enzyme loses its potassium dependence. Conversely, introduction of serine at the corresponding position in the potassium-independent enzyme from *P. vulgaris* endowed it with potassium sensitivity (Ajewole *et al.*, 2018 ▸).

Interestingly, it was also discovered that in the crystal structure of the D90E active-site mutant of the Class 1 EcAII enzyme there is a conserved zinc-binding site formed by Asp100, His197 and Asp200 (Borek *et al.*, 2014 ▸; Supplementary Fig. S8). This binding site seems to be quite specific despite the lack of an obvious biological context (Cerofolini *et al.*, 2019 ▸).

## 5. Class 3 asparaginases (formerly known as *R. etli*-type aspagainases)   

### 5.1. What do we know about *R. etli* asparaginases?   

The most mysterious class of l-asparaginases are the enzymes produced by *R. etli*. These soil bacteria belonging to the genus *Rhizobium* colonize the root nodules of the symbiotic legume *P. vulgaris* (common bean), in which they fix atmospheric nitrogen for its ultimate use in the form of 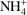 by the plant cells. On the other hand, organic carbon compounds (including amino acids) are supplied to the bacteria by the plant symbiont as the source of the energy necessary for nitrogen fixation (Huerta-Zepeda *et al.*, 1996 ▸). It has been reported that l-asparaginase activity in *R. etli* cultures was highest when the bacteria were grown on l-Asn as a nitrogen and carbon source, while the activity decreased in the presence of other carbon sources (glycerol, glucose or succinate; Huerta-Zepeda *et al.*, 1996 ▸). On the other hand, *R. etli* cultures grown on l-Gln showed only 10% of the asparaginase activity in the presence of l-Asn. Asparaginase activity in *R. etli* is not regulated by oxygen concentration, in contrast to EcAII, which is expressed under anaerobic conditions.

### 5.2. *R. etli* has two asparaginases: constitutive and inducible   

A report published in 1997 indicated that *R. etli*, similarly to other bacteria, also has two asparaginases: constitutive (thermostable) and inducible (thermolabile). The constitutive enzyme was stable at 50°C, while the inducible enzyme had a half-life of only 11 min at 50°C with two melting slopes (Huerta-Zepeda *et al.*, 1997 ▸). The two forms differ not only in thermostability but also in electrophoretic mobility: the constitutive protein migrated faster on native electrophoretic gels than the inducible protein. The activity (expression) of the thermolabile enzyme was positively regulated by its substrate l-Asn and was negatively regulated by carbon sources (Huerta-Zepeda *et al.*, 1997 ▸). Searching the European Nucleotide Archive revealed that the genome of *R. etli* CFN 42 (GCA_000092045.1; González *et al.*, 2006 ▸) encodes at least two l-asparaginases: one related to the *ansA* gene available under accession No. RHE_PE00350 and another deposited with accession No. RHE_CH01144 (Ensembl Bacteria repos­itory; Howe *et al.*, 2020 ▸). These data are in good agreement with previous reports (Huerta-Zepeda *et al.*, 1997 ▸). The *ansA* gene encoding the thermolabile enzyme is part of the *ansPAB* operon containing four adjacent ORFs: *ansR* (transcriptional repressor), *ansP* (l-asparagine permease), *ansA* (inducible l-asparaginase) and *ansB* (l-aspartase)^6^ (Ortuño-Olea & Durán-Vargas, 2000 ▸). The expression of the genes encoded by *ansPAB* is induced by the presence of l-Asn, and the products of this gene expression are responsible for the conversion of l-Asn to l-Asp and ammonia (thermolabile l-asparaginase) and the degradation of l-Asp to fumarate and ammonia in the next step (l-aspartase) (Huerta-Zepeda *et al.*, 1997 ▸). We abbreviate the two *R. etli* asparaginases as ReAIV (constitutive) and ReAV (inducible).

Very little is known about the constitutive *R. etli* asparaginase. It was only noted that ReAIV plays a minor role in the utilization of l-Asn as a nitrogen or carbon source (Huerta-Zepeda *et al.*, 1997 ▸). The level of sequence homology between ReAIV and ReAV is rather low, with 29.7% identity (Supplementary Fig. S9). Searching UniProt with *BLAST* (Johnson *et al.*, 2008 ▸; Altschul *et al.*, 1990 ▸) revealed that ReAIV has close homologs (50–80% sequence identity) in hundreds of bacterial species, predominantly in Proteobacteria (including Rhizobiales). It must be emphasized that the sequences of the constitutive/inducible *R. etli* enzymes are completely different from the bacterial-type (Class 1) or plant-type (Class 2) asparaginases, with identities of only about 13/14% (EcAI), 15/16% (EcAII) and 15/15% (EcAIII). A comprehensive search of the *R. etli* genome available via GenBank (NCBI reference number NC_007761.1; González *et al.*, 2006 ▸) using *BLAST* and the sequences of EcAI/II/III as targets revealed that this bizarre organism does not possess any type 1, type 2 or type 3 asparaginases. This justifies classifying the unique *R. etli*-type asparaginases as type 4 and type 5 and naming them ReAIV (constitutive) and ReAV (inducible) in this paper. Conversely, ReAIV and ReAV have no homologs in the *E. coli* genome.

### 5.3. Properties of the inducible *R. etli* asparaginase (ReAV)   

It was noticed early on that the sequence of ReAV is essentially different from the sequences of other microbial asparaginases and that ReAV has probably evolved independently of typical bacterial and fungal asparaginases (Borek & Jaskólski, 2001 ▸; Ortuño-Olea & Durán-Vargas, 2000 ▸). The lack of sequence similarity between ReAV and bacterial type 2 asparaginases opens the possibility of a different mechanism for asparagine degradation. It was found that ReAV has specific affinity for l-Asn and negligible affinity for l-Gln (Moreno-Enríquez *et al.*, 2012 ▸). Biophysical experiments show that recombinant ReAV is most active at alkaline pH (9.0–10.5), while its activity is about threefold lower at the physiological pH of 7.5 and is also reduced by about 27% at pH 11.0. It was also observed that at pH 9.0 the asparaginase activity of ReAV increased with temperature and reached a maximum at 50°C, but a further increase of temperature to 60°C resulted in enzyme inactivation. It was also found that the activity of ReAV was reduced in the presence of the reaction products, which acted as inhibitors: l-Asp (competitive) and 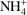 (noncompetitive). Divalent metal cations, such as Mn^2+^, Zn^2+^, Ca^2+^ and Mg^2+^, have also been reported as inhibitors (Moreno-Enríquez *et al.*, 2012 ▸), but in view of our own results this information may not be very reliable, at least with regard to some of the metals (Loch *et al.*, unpublished work). ReAV is also not reactive with d-Asn and β-aspartylhydroxamate, indicating a narrow substrate specificity for l-Asn. It was reported that ReAV shows a typical Michaelis–Menten profile, and the kinetic parameters (Supplementary Table S6) indicated that the enzyme has a lower affinity for l-Asn than EcAII.

*In silico* trials have been performed to model the structure of ReAV and predict its epitopes based on sequence analysis (Huerta-Saquero *et al.*, 2013 ▸). The computational model of ReAV has a completely different fold to known Class 1 or Class 2 asparaginases, as well as quite distant immunogenic properties. The computed ReAV structure has significant similarity to proteins with a β-lactamase/transpeptidase fold (Huerta-Saquero *et al.*, 2013 ▸).

A search of the UniProt database shows that most ReAV homologs appear in Firmicutes bacteria and in Proteobacteria, although some homologs are also present in eukarya, including the fungi Ascomycetes, often referred to as ‘green and blue molds’. The latter observation was noticed previously by Moreno-Enríquez *et al.* (2012 ▸), who suggested horizontal gene transfer between bacteria and fungi. Sequences similar to ReAV were found in fungi belonging to the genera *Aspergillus*, *Penicillium*, *Trichoderma*, *Colletotrichum* and *Fusarium* (50–63% identity). Some of these ubiquitous fungi are pathogenic to humans, but several of them also cause serious plant diseases.

## 6. Conclusions and outlook   

The simple amidohydrolysis at the side chain of asparagine has an amazing variety of biochemical realizations. It is a perplexing question as to why so many different enzymes have evolved to catalyze a very simple, almost trivial, reaction. This question does not have a plausible overarching answer, especially with the *R. etli* enzymes (and their evolution) very likely to complicate the situation even further. The typical bacterial asparaginases (types 1 and 2) found in Gram-negative bacteria serve different purposes in the cytoplasm (type 1) and in the periplasmic space (type 2). The low-affinity EcAI is expressed constitutively and plays a role in the physiological utilization of l-Asn as a nitrogen source. It degrades l-Asn when it is accumulated at an appropriate intracellular (cytoplasmic) concentration. The expression of the high-affinity EcAII is activated by anaerobiosis (by FNR and cyclic AMP receptor). Recent studies suggest that EcAII enables anaerobic respiration and is part of a metabolic pathway leading to the production of fumarate as a terminal electron acceptor. EcAII is localized in the periplasm to be able to utilize exogenous (extracellular) l-Asn to produce fumarate, as under anaerobic conditions transport of l-Asn from the cytoplasm to the periplasm is abolished (Srikhanta *et al.*, 2013 ▸). The role of Class 2 enzymes in plants is quite clear, particularly in symbiotic legumes. The atmospheric nitrogen assimilated in the roots of these plants is transported to metabolically active parts (shoots, leaves and flowers) in the form of l-Asn, where it is liberated by hydrolysis. Plant asparaginases are particularly important (and abundant) in seeds, where they have to supply NH_3_ for protein synthesis upon germination. Moreover, the second (isoaspartyl aminopeptidase) activity of these enzymes protects dormant seeds from the spontaneous accumulation of highly toxic β-aspara­gine dipeptides.

Although first detected in animal tissue almost 120 years ago, interest later focused on bacterial l-asparaginases with micromolar substrate affinity sufficient for successful application in the treatment of leukemia. However, even in bacteria another subtype of l-asparaginase is present, structurally similar but with a poor *K*_m_. The plant-specific enzymes that were discovered later are now known to have very close homologs in bacteria. While their different antigenic properties would make plant-type asparaginases excellent drug candidates, their *K*_m_ (millimolar) is too high for this purpose.

Structurally, bacterial-type (Class 1) and plant-type (Class 2) enzymes are completely different, but their active sites have intrinsically similar features, consisting of an abundance of threonine residues that function in a double-displacement ping-pong mechanism. Threonine as an unusual nucleophile was first indicated in the crystal structure of EcAII (Swain *et al.*, 1993 ▸) and was confirmed in this role by the crystal structure of archaeal proteasome (Löwe *et al.*, 1995 ▸). It would be tempting to speculate why threonine is the ‘nucleophile of choice’ in l-asparaginases. One reason might be that the nucleophile for asparagine hydrolysis does not have to be particularly strong, but it is of advantage if it has a structural feature (such as the methyl group) that would allow stereochemical control during the course of the reaction. However, we should be cautious with such speculation because the limited information that we have about *R. etli* asparaginases, for example about their tentative structural homology to β-lactamases, seems to indicate that in this class the nucleophile might be the more classical serine.

The asparaginases found in *R. etli*, a nitrogen-fixing bacterium living in symbiosis with legumes, are a relatively new addition to the asparaginase family. The full structural and mechanistic properties of these enzymes have yet to be elucidated. However, preliminary kinetic data indicate a submillimolar *K*_m_, just one order of magnitude shy of the range required for cancer therapy. It is thus possible that with suitable structure-based engineering the *R. etli*-type enzymes (which also have homologs in eukaryotes) could become the long-sought alternative antileukemics. Research on l-aspara­ginases has a fascinating history but possibly an even more exciting future.

## 7. Related literature   

The following references are cited in the supporting information for this article: Ajewole (2016 ▸), Anderson (2009 ▸), Chohan & Rashid (2013 ▸), Derst *et al.* (2000 ▸), Jaskólski *et al.* (2001 ▸), Kotzia & Labrou (2005 ▸, 2009 ▸), Lubkowski *et al.* (1996 ▸), Lubkowski, Wlodawer, Ammon *et al.* (1994 ▸), Lubkowski, Wlodawer, Housset *et al.* (1994 ▸), Maggi *et al.* (2017 ▸), Nakamura *et al.* (1971 ▸), Roberts (1976 ▸), Sanches *et al.* (2003 ▸), Steckel *et al.* (1983 ▸) and Tollersrud & Aronson (1992 ▸).

## Supplementary Material

Supplementary Tables and Figures. DOI: 10.1107/S2052252521006011/lz5050sup1.pdf

## Footnotes

1 Currently, *Erwinia chrysanthemi* is classified as *Dickeya dadantii.* However, in view of the widespread use of the historical name, we will also use it in our article.

2 It should be noted that the amino-acid sequence of Elspar (PDB entry 3eca) has four differences with respect to the sequence of EcAII from the UniProt database at positions 27 (V/A), 64 (N/D), 252 (S/T) and 263 (T/N). This is the reason why the sequence of the first EcAII model in the PDB is different from other depositions for the same protein.

3 To avoid confusing sequence numbers, the catalytic residues are labeled **^(n)^Xxx** according to their importance (*n* = 1, 2 …) in catalysis. Mapping of *n* onto concrete sequence numbers is provided in Supplementary Table S2. For plant-type asparaginases the system is similar, but the numbering starts with *n* = 100, as explained in Supplementary Table S4.

4 Detection of weak d-asparaginase activity may be controversial because optically pure d-Asn is virtually impossible to obtain.

5 In the literature, the same Greek letters (α, β) are used to denote both the subunits and secondary-structure elements. In most cases, in this review we qualify these symbols to avoid ambiguity.

6 It should be noted that in *E. coli* (and in most bacteria) the *ansA* gene encodes EcAI (the constitutive enzyme), while the *ansB* gene encodes EcAII (the inducible enzyme). Although the names of the genes are identical, in *R. etli* they encode completely different proteins.
