# Supplementary material for: Structural and biophysical aspects of l-asparaginases: a growing family with amazing diversity
Source: IUCrJ. 2021 Jun 30;8(Pt 4):514–31. doi: 10.1107/S2052252521006011 (PMC8256714; doi:10.1107/S2052252521006011)
Supplement: Supplementary file 1 [file m-08-00514-sup1.pdf]

IUCrJ

Volume 8 (2021)

Supporting information for article:

Structural and biophysical aspects of L-asparaginases: a growing family with amazing diversity

Joanna I. Loch and Mariusz Jaskolski

Supplementary Tables

Table S1. Examples of crystal structures of Class 1 (bacterial-type) L-asparaginases (and their mutants) and glutaminase-asparaginases available in the PDB.

	Abbreviation	gene	Uniprot	PDB IDs (examples)
**Class 1 type-1**				
Escherichia coli [1, 2]	EcAI	*ansA*	P0A962	6NXC, 6NXD, 2P2D, 2P2N, 2HIM
Pyrococcus furiosus [3]	PfAI	*PF2047*	Q8TZE8	4RA6, 4RA9, 5CBP, 4NJE, 5B5U, 5B74, 4Q0M
Pyrococcus horikoshii [4]	PhAI	*PH0066*	O57797	1WNF, 1WLS
Thermococcus kodakarensis [5]	TkAI	*TK1656*	Q5JIW4	5OT0
Vibrio cholerae [6]	VcAI	*VC_1995*	Q9KQK3	2OCD
Yersinia pestis [6]	YpAI	*ansA*	A0A384KLA7	3NTX
Cavia porcellus [7, 8]	CpAI	*ASPG*	H0W0T5	4R8K, 4R8L, 5DND, 5DNC, 5DNE
**Class 1 type-2**				
Campylobacter jejuni [6]	CjAII	*ansA*[1]	Q0PC96	3NXK
Helicobacter pylori [9]	HpAII	*ansA*[1]	Q9ZLB9	2WT4, 2WLT
Wolinella succinogenes [10, 11][2]	WsAII	*ansB*[3]	P50286	1WSA, 5K45, 5K4G, 5K4H, 6RUE, 6SYH, 6RUF, 5K3O
Erwinia chrysanthemi [12–17]	ErAII	*ansB*	P06608	5F52, 5I48, 5I3Z, 5I4B, 5HW0, 6PAE, 1JSL, 1JSR, 1HFK
Erwinia carotovora [4] [18, 19]	EwAII	*N/A*, lanS	Q6Q4F4, Q7WWK9	2HLN, 2JK0, 1ZCF
Escherichia coli [5] [2, 20–25]	EcAII	*ansB*	P00805	3ECA, 1JAZ, 1JJA, 6NX9, 6NX8, 6NX7, 6NX6, 6NXA
**Class 1 glutaminase-asparaginases (G-A)**				
Pseudomonas sp. 7A/ putida [6] [26–29]	PGA	*ansB*	P10182/Q88K39	4PGA, 3PGA, 1DJP, 1DJO, 6WZ4, 6WZ6, 6WYW, 6WZ8,
Acinetobacter glutaminasificans [30]	AGA	*ansB*	P10172	1AGX

[1] Described as product of *ansA* gene; however, the architecture of the active site and/or µM substrate (L-Asn) affinity (Table S2) suggest classification as a Class 1 type-2 enzyme.

[2] Two variants differing at position 121 (Ser/Pro) are reported.

[3] Ambiguous classification in Uniprot: "gene ansA synonym ansB".

[4] In the past classified as: *Pectobacterium atrosepticum, Pectobacterium atrosepticum SCRI1043, Pectobacterium carotovorum*.

[5] Includes *Escherichia coli* and *Escherichia coli K-12*.

[6] Strains *Pseudomonas sp.* (strain ATCC 29598 / 7A) or *Pseudomonas putida* (strain ATCC 47054 / DSM 6125 / NCIMB 11950 / KT2440).

Table S2. Unified nomenclature (residue numbers and **generic labels**) of residues involved in catalysis and important for substrate binding in different Class 1 L-asparaginases. Primed residues are contributed from the complementary subunit of the intimate dimer. A residue label in (front) superscript denotes difference from the consensus residue at this position specified in the column header.

**Unified nomenclature**[1]	$^{(1)}$**Thr**	$^{(2)}$**Tyr**	$^{(3)}$**Thr**	$^{(4)}$**Asp**	$^{(5)}$**Lys**	$^{(6)}$**Ser**	$^{(7)}$**Gln**	$^{(8)}$**Ala**	$^{(9)}$**Asn′**	$^{(10)}$**Glu′**
**Class 1 type-1**										
Escherichia coli	14	283′	91	92	163	60	^{Ser}61	^{Ser}117	246	-
Yersinia pestis	14	273′	91	92	163	60	^{Ser}61	^{Ser}117	246	-
Vibrio cholerae	14	284′	91	92	163	60	^{Ser}51	^{Ser}117	246	-
Pyrococcus furiosus	11	273′	83	84	154	52	^{Thr}53	^{Ser}109	^{Gly}236	-
Pyrococcus horikoshii	11	273′	83	84	154	52	^{Thr}53	^{Ser}109	^{Gly}236	-
Thermococcus kodakarensis	11	274′	83	85	156	54	^{Thr}55	^{Ser}111	^{Gly}236	-
Cavia porcellus	19	308′	116	117	188	85	^{Ser}86	142	272	-
**Class 1 type-2**										
Escherichia coli	12	25	89	90	162	58	59	114	248	283
Wolinella succinogenes	14	27	39	94	166	60	61	118	252	287
Helicobacter pylori	16	29	95	96	168	62	63	114	255	289
Erwinia chrysanthemi	15	29	95	96	168	62	63	120	^{Ser}254	^{Asp}287
Erwinia carotovora	15	29	95	96	168	62	63	120	^{Ser}254	^{Asp}287
Campylobacter jejuni	16	30	95	96	168	62	^{Ser}64	120	^{Ser}255	^{Asp}289
**Class 1 glutaminase-asparaginases (G-A)**										
Pseudomonas sp. 7A/ putida	20	34	100	101	173	97	^{Glu}68	^{Ser}125	^{Ser}258	294
Acinetobacter glutaminasificans	12	26	92	93	165	59	^{Glu}60	^{Ser}117	^{Ser}252	288

[1] Residues marked with ′ originate from a different subunit. $^{(2)}$Tyr originates form the same subunit as the catalytic triad $^{(1)}$Thr-$^{(2)}$Tyr-$^{(3)}$Thr in type-2 enzymes and in G-A, while it is donated by a different subunit in type-1 enzymes. Residues determining substrate preferences (L-Asn/L-Glu): $^{(9)}$Asn′ and $^{(10)}$Glu′, always come from the complementary subunit.

Table S3. Crystal structures of Class 2 (plant-type) asparaginases

**Class 2 (or type-3)**				
	**Abbreviation**	**gene**	**Uniprot**	**PDB IDs (examples)**
Escherichia coli [31–34]	EcAIII	*iaaA, ybiK*	P37595	2ZAK, 2ZAL, 3C17, 1T3M, 1JN9, 1K2X
Lupinus luteus [35]	LlAIII	*llA*	Q9ZSD6	2GEZ
Phaseolus vulgaris [36]	PvAIII(K)	*PvASPG1*	V7CU13	4PV3
Cavia porcellus [37]	CpAIII	*ASRGL1*	A0A286XYM2	4O48, 4O47
Homo sapiens [38–42]	HsAIII	*ASRGL1*	Q7L266	4PVR, 4PVS, 4PVP, 4PVQ, 4OSX, 4OSY, 4ZM9,

Table S4. Numbering of homologous residues and conserved structural elements in crystal structures of Class 2 asparaginases. A residue label in (front) superscript denotes difference from the consensus residue at this position specified in the column header.

**Unified nomenclature**	$^{(100)}$**Gly**	$^{(101)}$**Thr**	$^{(102)}$**Thr**	$^{(103)}$**Thr**	$^{(104)}$**Arg**	$^{(105)}$**Asp**	$^{(106)}$**Gly**	$^{(107)}$**Gly**	$^{(108)}$**Glu**	$^{(K)}$**His**	$^{(K)}$**Arg**	$^{(K)}$**Glu**	$^{(K)}$**Ser***	$^{(Na)}$**Asn**
Escherichia coli	178	179	197	230	207	210	231	233	234	119	207	234	^{Val}120	67
Homo sapiens	167	168	186	219	196	199	220	222	223	114	196	223	^{Cys}115	62
Lupinus luteus	192	193	211	243	221	224	244	246	247	118	221	247	^{Ile}118	66
Phaseolus vulgaris	195	196	214	246	224	227	247	249	250	117	224	250	118	65
Cavia porcellus	167	168	186	219	196	199	220	222	223	114	196	223	^{Cys}115	62

*Ser is present at this position in K-dependent enzymes, while in other enzymes Val, Cys or Ile was found in the corresponding position.

Table S5. *Rhizobium etli* (Class 3) asparaginases

**Class 3 (*R. etli*-type)**				
	**Abbrviation**	**gene**	**Uniprot**	**PDB IDs (examples)**
Rhizobium etli	ReAIV	*RHE_CH01144*	Q2KB35	-
Rhizobium etli	ReAV	*ansA*	Q2K0Z2	-

Table S6. Kinetic parameters of selected asparaginases reviewed in this paper. The kinetic parameters are specified for L-Asn substrate.

	K_M [M]	k_{cat} [s^{-1}]	k_{cat}/K_M [$M^{-1}s^{-1}$]	tested pH, (maximum)	tested temp., Tm, (optimal)
**Class 1 type-1**					
Escherichia coli	1.2×10^{-3}	*no data*	*no data*	*no data*	*no data*
Pyrococcus furiosus [43]	12×10^{-3}	870	72 500	4.0-10.5, (9.0)	37-95°C, 85°C, (*no data*)
Thermococcus kodakarensis [44]	5.5×10^{-3}	1397	254 000	6.0-11.0, (9.5)	30-95°C, *no data*, (85°C)
Cavia porcellus [7]	57.7×10^{-6}	38.6	800 000	*no data*	*no data*
**Class 1 type-2**					
Helicobacter pylori [9]	19×10^{-6}	*no data*	*no data*	*no data*	*no data*
Wolinella succinogenes (S121)[10]	21.7×10^{-6}	97.8	4 500 000	*no data*	*no data*
Wolinella succinogenes (P121)[10]	23.6×10^{-6}	86.7	3 700 000	*no data*	*no data*
Erwinia chrysanthemi [45]	58×10^{-6}	23 800	411 800	*no data*	30-70°C, 46.4°C, (*no data*)
Erwinia carotovora [46]	85×10^{-6}	31 400	369 000 000	4-10, (6.5-9.0)	*no data*
Escherichia coli [47–49]	35×10^{-6}	29	830 000	3-11, (6.0-8.0)	40-80°C, 49°C, (*no data*)
**Class 1 glutaminase-asparagniases**					
Pseudomonas sp. 7A / putida [50]	4.4×10^{-6}	*no data*	*no data*	4-10, (not detected)	*no data*
Acinetobacter glutaminasificans [51]	40×10^{-6}	*no data*	*no data*	*no data*	*no data*
**Class 2 (or type-3)**					
Escherichia coli [52]	3.90×10^{-3}	0.28	71.8	*no data*	*no data*, 59.2°C, (*no data*)
Homo sapiens [42, 53]	2.09×10^{-3}	3.19	1 520	3-10, (5.5-6.0)	*no data*, 70°C, (*no data*)
Lupinus luteus [52]	4.90×10^{-3}	0.32	66.7	*no data*	*no data*
Phaseolus vulgaris [54][1]	3.43×10^{-3}	37.4	10 903	5.5-10.0 (7.5)	5-95°C,48.5/48.0°C, (*no data*)
Cavia porcellus [37]	2.24×10^{-3}	3.95	1 760	*no data*	*no data*
**Class 3 (*R.etli*-type)**					
Rhizobium etli (inducible enzyme) [55][2]	8.9×10^{-3}	106	12 000	7.5-11.0	50-60°C, *no data*, (37°C)

[1] Determined in the presence of potassium cations.

[2] Recently determined kinetic parameters indicate that the K_M is lower, in the range of ~4×10^{-4} [M] (Loch & Sliwiak, unpublished).

Supplementary Figures

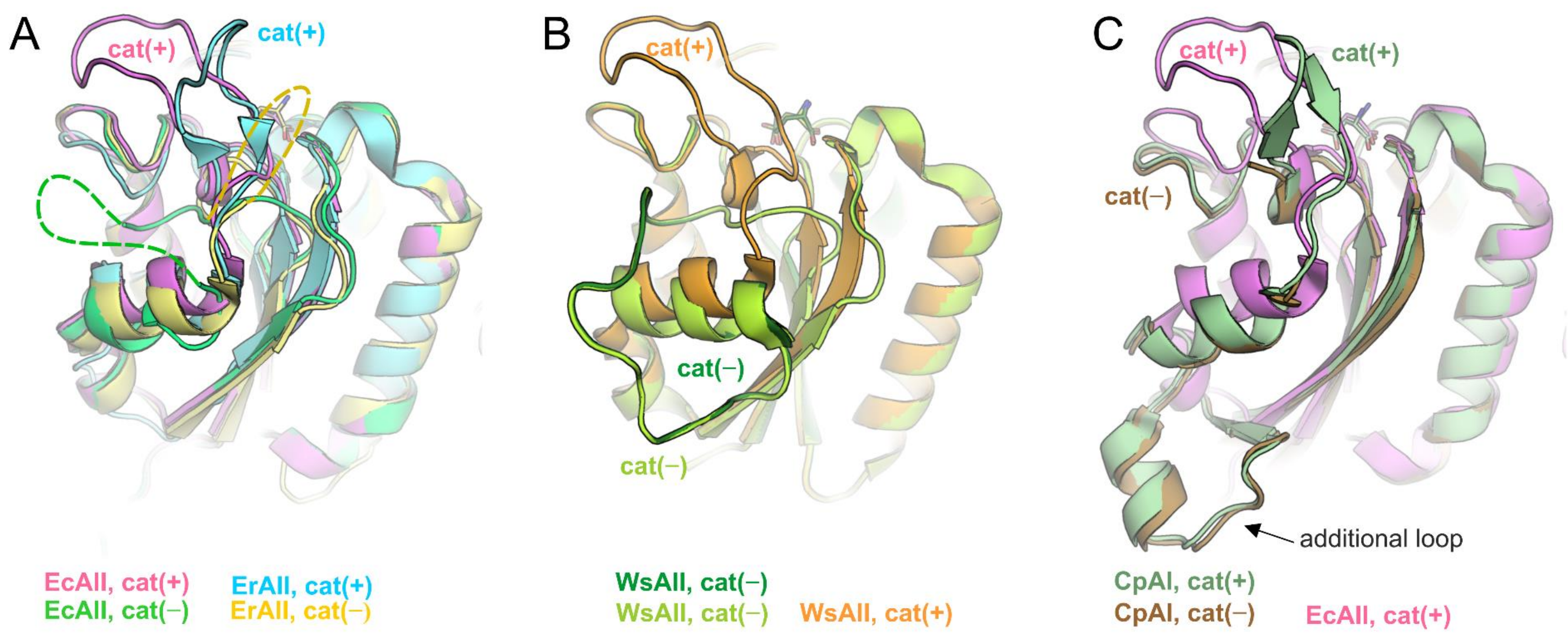

Fig. S1. Comparison of conformational states of FGE in different Class 1 asparaginases. (A) EcAII with the state of FGE in cat(−) (green, PDB ID 6v23) and (in the presence of L-Asp) cat(+) (pink, PDB ID 6pab), ErAII (in the presence of L-Gln) in cat(−) (yellow, PDB ID 1hfw), PhAI (in the absence of ligands) in cat(+) (cyan, PDB ID 1wsl). (B) WsAII (in the presence of L-Asp) in cat(+) (orange, PDB ID 5ko3), WsAII (in the presence of L-Asp) in cat(−) (light green, PDB ID 5k4g) and (in the presence of L-Gln) in cat(+) (dark green, PDB ID 5k45). (C) CpAI (in the presence of L-Asp) in cat(+) (pale green, PDB ID 4r8l) and (in the absence of ligands) in cat(−) (brown, PDB ID 4r8k). The CpAI model was superposed on EcAII in the cat(+) state (pink, PDB ID 6pab). The additional loop in the N-terminal domain of the mammalian enzymes is marked by arrow. Green and yellow dashed lines in panel A mark a missing (disordered) fragment of the FGE in EcAII and ErAII in the cat(−) state.

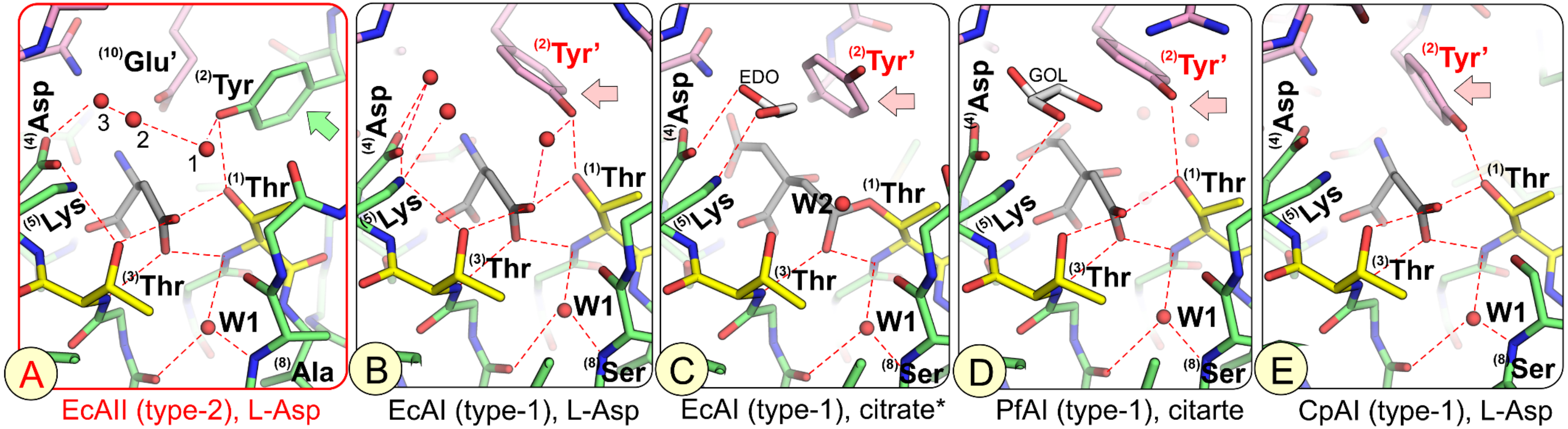

Fig. S2. Comparative analysis of the of active site structure in Class 1 L-asparaginases. (A) L-Asp bound in the active site of the model bacterial type-2 L-asparaginase EcAII (PDB ID 3eca). (B) L-Asp bound in the active site of EcAI (PDB ID 4nje). **(2)Tyr′**, which activates **(1)Thr**, is contributed by the complementary protomer (marked by **′**). (C) A similar situation is observed in EcAI trapped in the acyl-enzyme intermediate state in the presence of citrate ions; however, in this case **(2)Tyr′** is rotated away from **(1)Thr** (PDB ID 6nxc). (D) Citrate ion in the active site of the type-1 enzyme from *P. furiosus*; **(2)Tyr′** from the other protomer activates **(1)Thr**, just as in (B). (E) L-Asp bound in the active site of the mammalian (*C. porcellus*) type-1 enzyme (PDB ID 4r8l). W1 and W2 are conserved in all bacterial enzymes, while waters 1, 2 and 3 are typical for bacterial type-2 enzymes. Fat green arrow marks the position of **(2)Tyr** in bacterial type-2 enzymes, while fat pink arrows mark the position of **(2)Tyr′** in type-1 enzymes. In (C) and (D), in close vicinity of **(2)Tyr′** small ligands such as ethylene glycol (EDO) or glycerol (GOL) were identified.

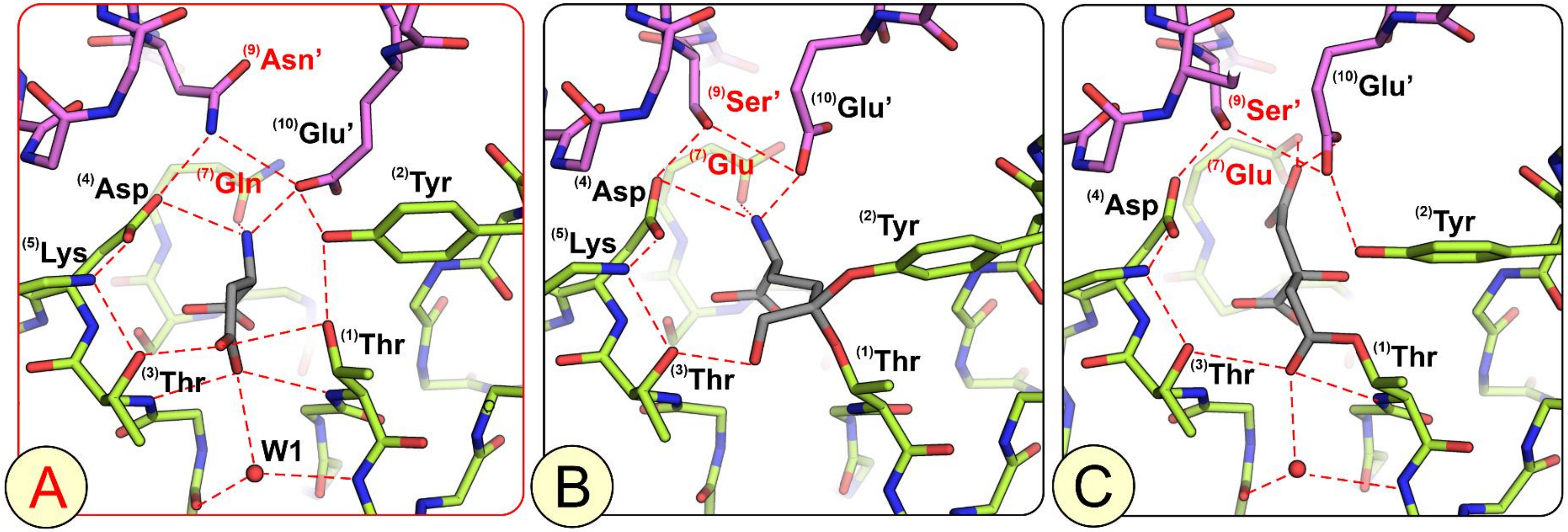

Fig. S3. Comparison of the active site structure in EcAII and in selected glutaminase-asparaginases (G-A) with FGE in cat(+) conformation. Chains colored green and violet belong to different subunits. To date, there is no crystal structure of a complex between G-A and L-Gln/L-Glu bound in the active site, with FGE in cat(+) conformation. (A) EcAII (PDB ID 3eca) with L-Asp in the active site. Residues determining L-Asn affinity, **(7)Gln** and **(9)Asn′**, are marked in red. (B) G-A from *Pseudomonas sp. 7A* (PDB ID 1djp) with the inhibitor L-DON covalently bound to **(1)Thr** and **(2)Tyr**. (C) G-A from *Pseudomonas putida* (PDB ID 6wyw) with citrate covalently bound to **(1)Thr**. In G-A structures, similarly to bacterial type-2 asparaginases, **(2)Tyr**, which covers the active site, is located in the same subunit as the catalytic threonines.

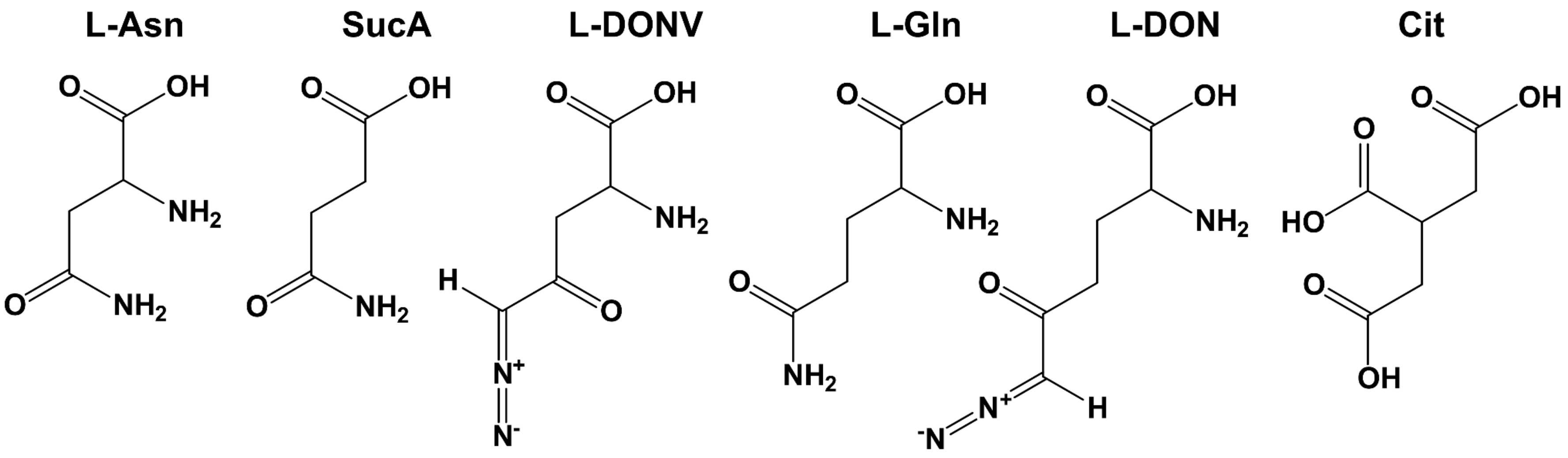

Fig. S4. Structures of L-asparaginase substrates and inhibitors: L-Asn (L-asparagine), SucA (succinic monoamide), L-DONV (5-diazo-4-oxo-L-norvaline), L-Gln (L-glutamine), L-DON (6-diazo-5-oxo-L-norleucine), Cit (citric acid).

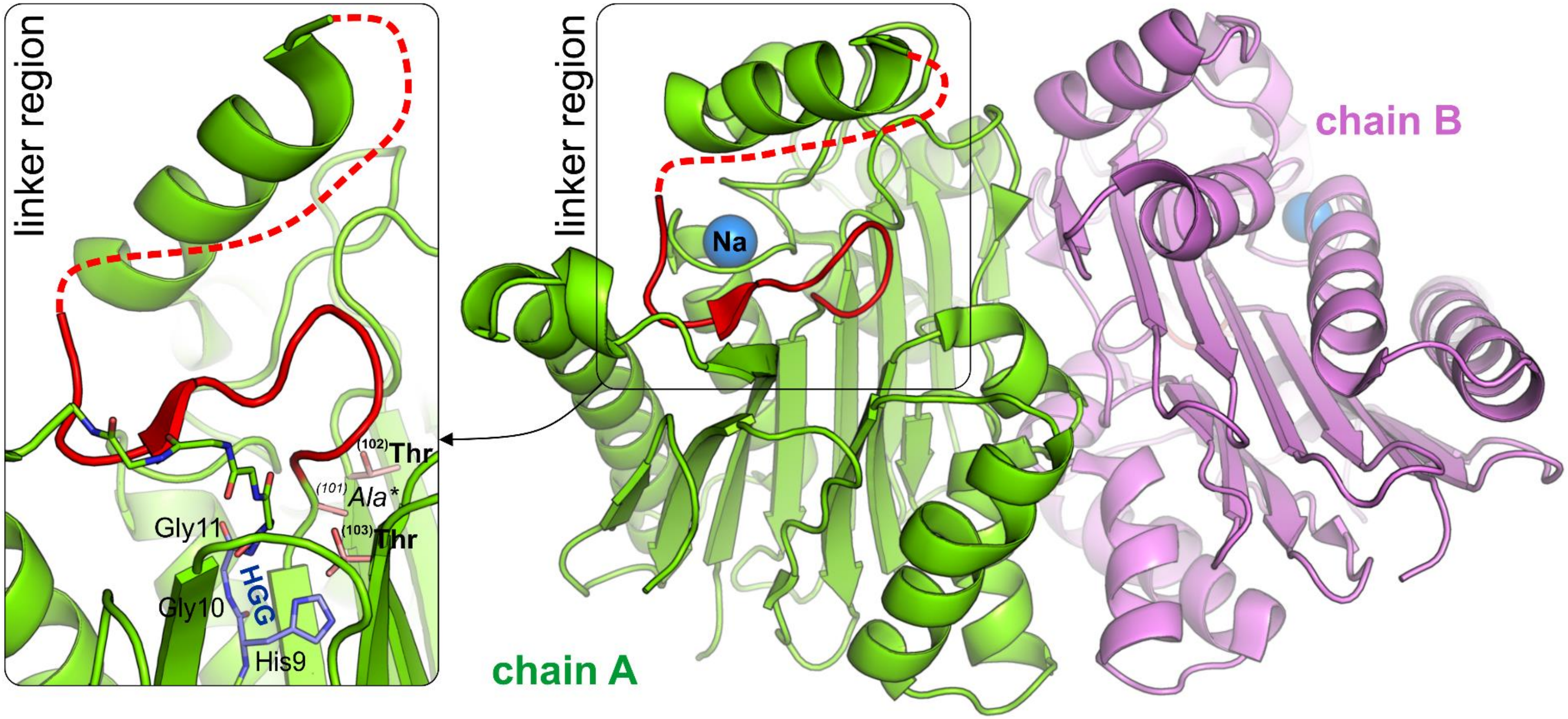

Fig. S5. Crystal structure of uncleaved precursor of a typical Class 2 L-asparaginase, EcAIII (PDB ID 3c17), with T179A mutation, comprised of chain A (green) and chain B (violet). The linker region (red) is only partially visible in the crystal structure (the missing part is marked with red dashed line). The inset represents a zoom in view of the linker region and the active site with the catalytic triad **(101)Thr-*(102)Ala**-(103)Thr** (**(102)Thr** replaced with ***(102)Ala****) and the HGG loop (blue).

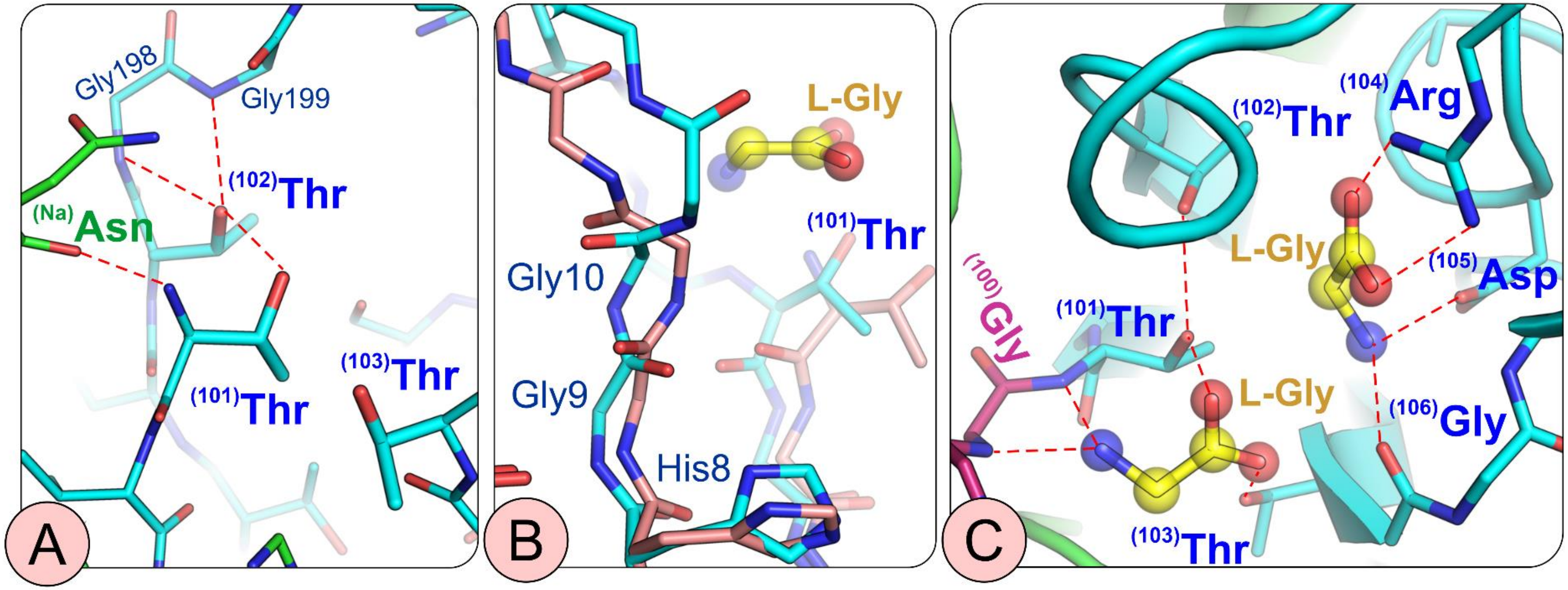

Fig. S6. Detailed view on the active site of Class 2 L-asparaginases. (A) H-bonds between **(101)Thr-(102)Thr** and carbonyl oxygen of **(Na)Asn** in EcAIII (PDB ID 2zal). (B) Differences in conformation of the HGG loop (His8, Gly9, Gly10) in the cleaved (PDB ID 4o0g, salmon) and uncleaved (PDB ID 4osx, cyan) HsAIII. (C) Glycine molecules (yellow) bound in uncleaved HsAIII (PDB ID 4osx); a fragment of the linker region is colored dark pink.

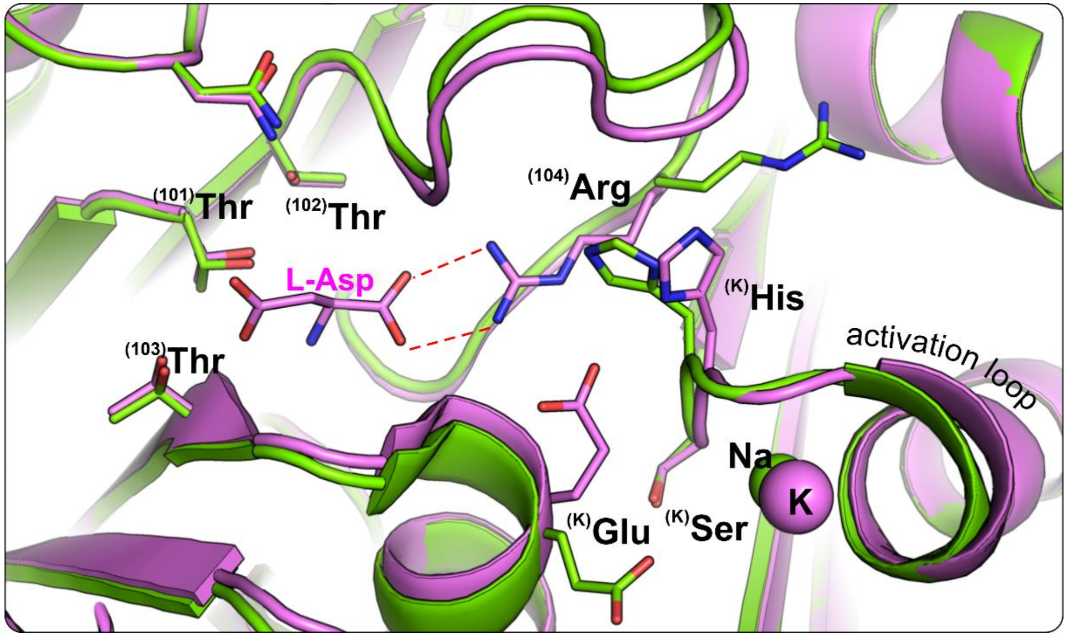

Fig. S7. Potassium-dependent asparaginase from *P. vulgaris* in ON (PDB ID 4pu6, violet) and OFF (PDB ID 4pv3, green) states. In the absence of K^+ ions, the activation loop coordinates Na^+ ions.

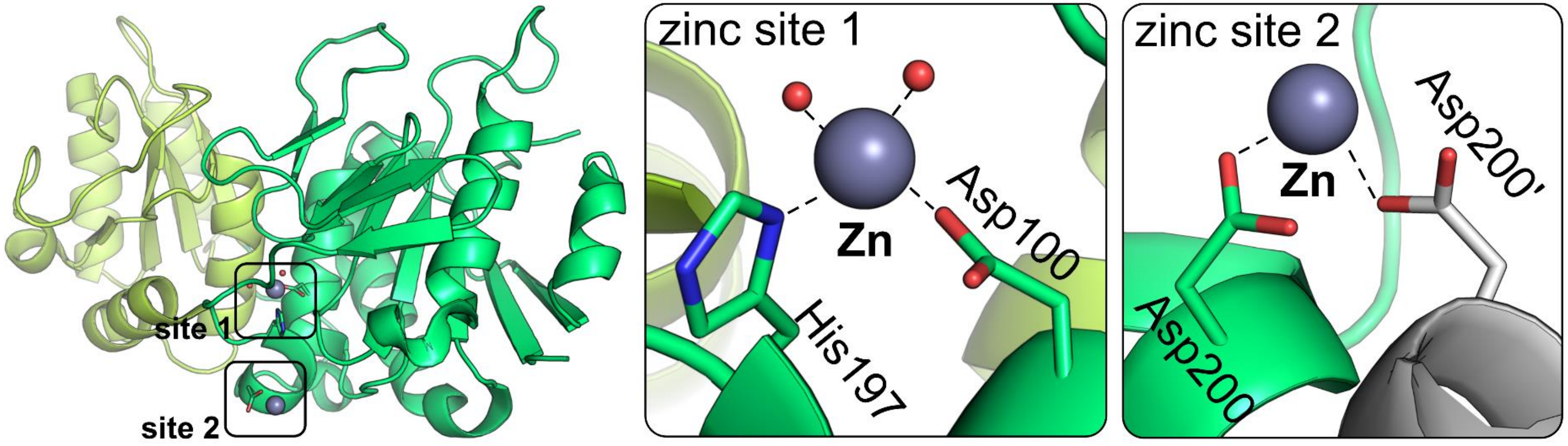

Fig. S8. Zinc binding sites in the EcAII structure PDB ID 1jaz. The N-terminal domain is colored dark green, while the C-terminal domain is colored light green. The zinc binding site 2 is composed of Asp200 and Asp200’ from a symmetry-related molecule (gray).

```
ReAIV  -MTNPVTVEVTRGLLVESRHRGAVAVVDGDGKLFFSLGDIDTAVFPRSACKAMQALPLVE 59
ReAV   MTPSEDFVVTDRGGIVENSHRVHAAVVDAKGRLLYALGNPTRMTLARSAAKPAQALAILE 60
          .   * . ** :**. **  .****..*:*:::**:    .: ***.*  *** ::*

ReAIV  SGAADAYGFGDKELALACASHNGEEEHVALAASMLSRAGRNVEALECGAHWSMNQKVLIQ 119
ReAV   TEGVAGYGFDDADIALMCASHSSEDRHIARTRAMLSKIKAEEADLRCGGHPSLSEMVNRS 120
       : .. .***.* ::** ****..*:.*:* : :***:   :   *.**.* *:.: *  .

ReAIV  QARSLDAPTALHNNCSGKHAGFICACCHRDIDPKGYVGYEHPLQVEIRAVMERLTGAVLG 179
ReAV   WIKQDFIPTAVCSNCSGKHVGMLAGARAIGAGTDGYHLPDHPMQGRVKRTVAELCDLDAG 180
         :.   ***: .******.*::...   . . .**   :**:* .:: .: .* .   *

ReAIV  AESCGTDGCSIPTYAMPLRNLAHGFARMATGTGLE------PLRAKASRRLIEACMAEPF 233
ReAV   DVEWGTDGCNLPTPAFPLDRLGRIYAKLASAADGSDAGEGQSTRCAALAHIFRAMARHPE 240
         . *****.:** *:** .*.: :*::*:.:. .        *. *  :::.*   .*

ReAIV  YVAGSGRACTKLMQIAPGRIFVKTGAEGVFCAAIP----------EKGIGISLKSEDGAT 283
ReAV   MVAGEGRYCTMLMRAFDGALVGKLGADASYAIGVRASDATRQLGTDGALGISVKIEDGNL 300
        ***.** ** **:   * :. * **:. :. .:           : .:***:* ***

ReAIV  RAAEAMVAATLARFFETEETVHAALMAFAAMPMRNWNGIHVGDIRATSVFSA-------- 335
ReAV   EMLYAVVTELLERLGIGSPDVRSQLASFHHPQRVNTMGVTTGGVSFPFKLRGSKSNVDDP 360
       .   *:*:  * *:   .  *:: * :*      *  *: .*.:     : .

ReAIV  -------
ReAV   RLAAVAR 367
```

Fig. S9. Sequence alignment of *R. etli* constitutive asparaginase (ReAIV, Uniport Q2KB35) and *R. etli* inducible asparaginase (ReAV, Uniprot Q2K0Z2). Green color marks identical residues.

References

[1] Yun, M. K., Nourse, A., White, S. W., Rock, C. O. & Heath, R. J. (2007). *J. Mol. Biol.* **369**, 794–811.
[2] Lubkowski, J., Chan, W. & Wlodawer, A. (2019). *Sci. Rep.* **9**, 1–14.
[3] Tomar, R., Sharma, P., Srivastava, A., Bansal, S., Ashish & Kundu, B. (2014). *Acta. Cryst.* **D70**, 3187–3197.
[4] Yao, M., Yasutake, Y., Morita, H. & Tanaka, I. (2005). *Acta. Cryst.* **D61**, 294–301.
[5] Guo, J., Coker, A. R., Wood, S. P., Cooper, J. B., Chohan, S. M., Rashid, N. & Akhtar, M. (2017). *Acta. Cryst.* **D73**, 889–895.
[6] Anderson, W. (2009). *Infect. Disord. Drug. Targets.* **9**, 507–17.
[7] Schalk, A. M., Nguyen, H. A., Rigouin, C. & Lavie, A. (2014). *J. Biol. Chem.* **289**, 33175–33186.
[8] Schalk, A. M., Antansijevic, A., Caffrey, M. & Lavie, A. (2016). *J. Biol. Chem.* **291**, 5088–50100.
[9] Dhavala, P. & Papageorgiou, A. C. (2009). *Acta. Cryst.* **D65**, 1253–1261.
[10] Nguyen, H. A., Durden, D. L. & Lavie, A. (2017). *Sci. Rep.* **7**, 1–14.
[11] Lubkowski, J., Palm, G. J., Gilliland, G. L., Derst, C., Röhm, K. H. & Wlodawer, A. (1996). *Eur. J. Biochem.* **241**, 201–207.
[12] Miller, M., Rao, J. K. M., Wlodawer, A. & Gribskov, M. R. (1993). *FEBS Lett.* **328**, 275–9.
[13] Aghaiypour, K., Wlodawer, A. & Lubkowski, J. (2001). *Biochim. Biophys. Acta, Protein Struct. Mol. Enzymol.* **1550**, 117–128.
[14] Nguyen, H. A., Su, Y. & Lavie, A. (2016). *Biochemistry* **55**, 1246–1253.
[15] Aghaiypour, K., Wlodawer, A. & Lubkowski, J. (2001). *Biochemistry* **40**, 5655–5664.
[16] Nguyen, H. A., Su, Y. & Lavie, A. (2016). *J. Biol. Chem.* **291**, 17664–17676.
[17] Lubkowski, J., Dauter, M., Aghaiypour, K., Wlodawer, A. & Dauter, Z. (2003). *Acta. Cryst.* **D59**, 84–92.
[18] Kravchenko, O. V., Kislitsin, Y. A., Popov, A. N., Nikonov, S. V. & Kuranova, I. P. (2008). *Acta. Cryst.* **D64**, 248–256.
[19] Papageorgiou, A. C., Posypanova, G. A., Andersson, C. S., Sokolov, N. N. & Krasotkina, J. (2008). *FEBS J.* **275**, 4306–4316.
[20] Lubkowski, J. & Wlodawer, A. (2019). *Protein Sci.* **28**, 1850–1864.
[21] Borek, D., Kozak, M., Pei, J. & Jaskolski, M. (2014). *FEBS J.* **281**, 4097–40111.
[22] Sanches, M., Barbosa, J. A. R. G., De Oliveira, R. T., Neto, J. A. & Polikarpov, I. (2003). *Acta. Cryst.* **D59**, 416–422.
[23] Jaskólski, M., Kozak, M., Lubkowski, J., Palm, G. & Wlodawer, A. (2001). *Acta. Cryst.* **D57**, 369–377.
[24] Palm, G. J., Lubkowski, J., Derst, C., Schleper, S., Röhm, K. H. & Wlodawer, A. (1996). *FEBS Lett.* **390**, 211–216.
[25] Swain, A. L., Jaskolski, M., Housset, D., Rao, J. K. M. & Wlodawer, A. (1993). *Proc. Natl. Acad. Sci. USA* **90**, 1474–1478.
[26] Ortlund, E., Lacount, M. W., Lewinski, K. & Lebioda, L. (2000). *Biochemistry* **39**, 1199–1204.
[27] Jakob, C. G., Lewinski, K., LaCount, M. W., Roberts, J. & Lebioda, L. (1997). *Biochemistry* **36**, 923–931.
[28] Lubkowski, J., Wlodawer, A., Swain, A. L., Ammon, H. L. & Copeland, T. D. (1994). *Biochemistry* **33**, 10257–10265.
[29] Strzelczyk, P., Zhang, D., Dyba, M., Wlodawer, A. & Lubkowski, J. (2020). *Sci. Rep.* **10**, 1–16.
[30] Lubkowski, J., Wlodawer, A., Housset, D., Weber, I. T., Ammon, H. L., Murphy, K. C. & Swain, A. L. (1994). *Acta. Cryst.* **D50**, 826–832.
[31] Michalska, K., Borek, D., Hernändez-Santoyo, A. & Jaskolski, M. (2008). *Acta. Cryst.* **D64**, 309–320.
[32] Michalska, K., Brzezinski, K. & Jaskolski, M. (2005). *J. Biol. Chem.* **280**, 28484–28491.
[33] Michalska, K., Hernandez-Santoyo, A. & Jaskolski, M. (2008). *J. Biol. Chem.* **283**, 13388–13397.
[34] Prahl, A., Pazgier, M., Hejazi, M., Lockau, W. & Lubkowski, J. (2004). *Acta. Cryst.* **D60**, 1173–1176.
[35] Michalska, K., Bujacz, G. & Jaskolski, M. (2006). *J. Mol. Biol.* **360**, 105–116.
[36] Bejger, M., Imiolczyk, B., Clavel, D., Gilski, M., Pajak, A., Marsolais, F. & Jaskolski, M. (2014). *Acta. Cryst.* **D70**, 1854–1872.
[37] Schalk, A. M. & Lavie, A. (2014). *Biochemistry* **53**, 2318–2328.

[38] Nomme, J., Su, Y., Konrad, M. & Lavie, A. (2012). *Biochemistry* **51**, 6816–6826.
[39] Su, Y., Karamitros, C. S., Nomme, J., McSorley, T., Konrad, M. & Lavie, A. (2013). *Chem. Biol.* **20**, 533–540.
[40] Li, W., Irani, S., Crutchfield, A., Hodge, K., Matthews, W., Patel, P., Zhang, Y. J. & Stone, E. (2016). *Biochemistry* **55**, 960–969.
[41] Li, W., Cantor, J. R., Yogesha, S. D., Yang, S., Chantranupong, L., Liu, J. Q., Agnello, G., Georgiou, G., Stone, E. M. & Zhang, Y. (2012). *ACS Chem. Biol.* **7**, 1840–1847.
[42] Nomme, J., Su, Y. & Lavie, A. (2014). *J. Mol. Biol.* **426**, 2471–2485.
[43] Bansal, S., Gnaneswari, D., Mishra, P. & Kundu, B. (2010). *Biochemistry (Moscow)* **75**, 375–381.
[44] Chohan, S. M. & Rashid, N. (2013). *J. Biosci. Bioeng.* **116**, 438–443.
[45] Kotzia, G. A. & Labrou, N. E. (2009). *FEBS J.* **276**, 1750–1761.
[46] Kotzia, G. A. & Labrou, N. E. (2005). *J. Biotechnol.* **119**, 309–323.
[47] Derst, C., Henseling, J. & Röhm, K.-H. (2000). *Protein Sci.* **9**, 2009–2017.
[48] Nakamura, N., Morikawa, Y., Fujio, T. & Tanaka, M. (1971). *Agric. Biol. Chem.* **35**, 219–225.
[49] Maggi, M., Mittelman, S. D., Parmentier, J. H., Colombo, G., Meli, M., Whitmire, J. M., Merrell, D. S., Whitelegge, J. & Scotti, C. (2017). *Sci. Rep.* **7**, 1–16.
[50] Roberts, J. (1976). *J. Biol. Chem.* **251**, 2119–2123.
[51] Steckel, J., Roberts, J., Philips, F. S. & Chou, T. C. (1983). *Biochem. Pharmacol.* **32**, 971–977.
[52] Borek, D., Michalska, K., Brzezinski, K., Kisiel, A., Podkowinski, J., Bonthron, D. T., Krowarsch, D., Otlewski, J. & Jaskolski, M. (2004). *Eur. J. Biochem.* **271**, 3215–3226.
[53] Tollersrud, O. K. & Aronson, N. N. (1992). *Biochem. J.* **282**, 891–897.
[54] Ajewole, E. O. (2016). *PhD thesis*.
[55] Ortuño-Olea, L. & Durán-Vargas, S. (2000). *FEMS Microbiol. Lett.* **189**, 177–182.
